# Deciphering the nature of the coral–*Chromera* association

**DOI:** 10.1038/s41396-017-0005-9

**Published:** 2018-01-10

**Authors:** Amin R Mohamed, Vivian R Cumbo, Saki Harii, Chuya Shinzato, Cheong Xin Chan, Mark A Ragan, Nori Satoh, Eldon E Ball, David J Miller

**Affiliations:** 1 ARC Centre of Excellence for Coral Reef Studies, James Cook University, Townsville, 4811 QLD Australia; 2 Comparative Genomics Centre and Department of Molecular and Cell Biology, James Cook University, Townsville, 4811 QLD Australia; 3 Zoology Department, Faculty of Science, Benha University, Benha, 13518 Egypt; 4 AIMS@JCU, Australian Institute of Marine Science, Department of Molecular and Cell Biology, James Cook University, Townsville, 4811 QLD Australia; 5 Department of Biological Sciences, Macquarie University, Sydney, NSW 2109 Australia; 6 Sesoko Station, Tropical Biosphere Research Center, University of the Ryukyus, 3422 Sesoko, Motobu, Okinawa 905-0227 Japan; 7 Marine Genomics Unit, Okinawa Institute of Science and Technology Graduate University, Onna, Okinawa 904-0412 Japan; 8 Institute for Molecular Bioscience, The University of Queensland, Brisbane, QLD 4072 Australia; 9 Division of Ecology and Evolution, Research School of Biology, Australian National University, Acton, ACT 2601 Australia; 10 Present Address: Atmosphere and Ocean Research Institute, The University of Tokyo, 5-1-5, Kashiwanoha, Kashiwa-shi, Chiba 277-8564 Japan

## Abstract

Since the discovery of *Chromera velia* as a novel coral-associated microalga, this organism has attracted interest because of its unique evolutionary position between the photosynthetic dinoflagellates and the parasitic apicomplexans. The nature of the relationship between *Chromera* and its coral host is controversial. Is it a mutualism, from which both participants benefit, a parasitic relationship, or a chance association? To better understand the interaction, larvae of the common Indo-Pacific reef-building coral *Acropora digitifera* were experimentally infected with *Chromera*, and the impact on the host transcriptome was assessed at 4, 12, and 48 h post-infection using Illumina RNA-Seq technology. The transcriptomic response of the coral to *Chromera* was complex and implies that host immunity is strongly suppressed, and both phagosome maturation and the apoptotic machinery is modified. These responses differ markedly from those described for infection with a competent strain of the coral mutualist *Symbiodinium*, instead resembling those of vertebrate hosts to parasites and/or pathogens such as *Mycobacterium tuberculosis*. Consistent with ecological studies suggesting that the association may be accidental, the transcriptional response of *A. digitifera* larvae leads us to conclude that *Chromera* could be a coral parasite, commensal, or accidental bystander, but certainly not a beneficial mutualist.

## Introduction

Although the association between photosynthetic algae of the genus *Symbiodinium* and corals has been known for many years, recent work has shown that several lineages of apicomplexans are also associated with corals (e.g., [1–4]). Ribosomal small subunit RNA (rRNA) sequences previously misidentified as of bacterial origin in fact originate from eight distinct novel apicomplexan-related lineages (ARLs), a subset of which are associated with corals [4]. Two of these ARLs, *Chromera velia* and *Vitrella brassicaformis*, are alveolates of the phylum Chromerida [2]. *Chromera* is the closest known photosynthetic relative of the apicomplexan parasites, but is also related to the photosynthetic dinoflagellates including the coral mutualist *Symbiodinium* [2]*.* *Chromera* was initially isolated from the scleractinian corals *Plesiastrea versipora* from Sydney harbor and *Leptastrea purpurea* from One Tree Island on the Great Barrier Reef (GBR), Australia [2]. More recently, Cumbo et al. [5] isolated *Chromera* from another scleractinian coral, *Montipora digitata* (Acroporidae), from Nelly Bay, Magnetic Island, in the inner central region of the GBR, and established that this strain of *Chromera* can infect larvae of both *Acropora digitifera* and *A. tenuis*. However, *Chromera* is clearly not limited to Australian corals, as it has also been isolated from *Agaricia agaricites* in the Caribbean [6], and identified in sequence data from Curaçao [4, 7]. While the photosynthetic capacity of *Chromera* raises the possibility of it having a beneficial relationship with corals, it is also possible that the interaction may not be mutualistic, given that the majority of apicomplexans are parasites. Little is known about the coral response to pathogens, but, in general, host transcriptional responses to potential mutualists differ markedly from those to parasites [8]. While the innate immune repertoire of corals is surprisingly complex and vertebrate-like [9], to date few studies have addressed immune responses at the transcriptomic level [10–15]. However, the response of *Acropora* to a competent *Symbiodinium* strain (a strain able to infect coral larvae and establish a mutualistic relationship) is both subtle [16] and transient [17], whereas non-competent strains (that cannot establish a mutualistic relationship) trigger immune responses [16]. The host transcriptional response may, therefore, provide clues as to the nature of the interaction between coral and *Chromera*.

To investigate the coral response to *Chromera* infection, gene expression levels in *Chromera*-infected *A. digitifera* larvae were compared to uninfected larval controls at 4, 12, and 48 h post-infection by mapping Illumina RNA-Seq reads onto the *A. digitifera* transcriptome [18]. In contrast to the response to a competent *Symbiodinium* strain, the response to *Chromera* resembled that to a non-competent strain in taking place on a longer timescale and involving differential expression of larger numbers of genes. The coral response to *Chromera* infection was complex, involving modulation of the endocytic pathway and suppression of both immunity and apoptosis, and resembles the vertebrate responses to pathogens and some parasites. The possibility that *Chromera* has a beneficial relationship with coral hosts requires reevaluation in light of the data presented here.

## Materials and methods

### *Chromera* culture

*Chromera velia* CCMP2878 was used in this experiment and was originally isolated from *Plesiastrea versipora* (Faviidae) from Sydney harbor [2]. Cultures were grown in axenic f/2 medium (G0154, Sigma-Aldrich) [19] and maintained at 25 °C under a 12/12-h light/dark cycle before they were used for infection of coral larvae.

### Coral larvae and *Chromera* infection experiment

Approximately 700 planula larvae (6 day post-fertilization) were distributed into each of six 1 L plastic containers containing 700 ml of 0.2 μm FSW, giving three replicates each of uninfected and *Chromera*-infected larvae. Cultured *Chromera* were washed three times in 0.2 μm FSW and added at a density of 5 × 10^3^ cells/ml. Containers were held at 26 °C under fluorescent lamps that provided light (86 ± 2 μmol photon/ m^2^/s measured at the surface) on a 12/12-h light/dark cycle. At 4, 12 and 48 h post-infection, ~150 larvae from each replicate were washed in 0.2 μm FSW (ensuring as little liquid carry-over as possible), snap frozen, and stored at −80 °C until further treatment. Total RNA from these larvae was isolated as described by Mohamed et al. [17] using TRIzol® reagent (Ambion Life Technologies, Austin, TX, USA) according to manufacturer’s instructions. RNA was dissolved in 40 μl of RNase-free water and stored at −80 °C. RNA was quantitated and its integrity assessed using a NanoDrop ND-1000 spectrometer (Wilmington, DE, USA) and Agilent 2100 Bioanalyzer (Santa Clara, CA, USA).

### Sequencing and RNA-Seq data analysis

RNA-Seq data were obtained and analyzed as previously described by Mohamed et al.[17]. In brief, Illumina reads were mapped onto the *A. digitifera* transcriptome assembly v1.0 [18] using BOWTIE [20] with default parameters, then transcript counts in each sample were obtained using RSEM software version 1.1.17 [21]. Differential gene expression was inferred based on the mapping counts using edgeR [22]. *Chromera*-infected samples were compared to control samples at each of the three time points; 4, 12, and 48 h post-infection. *P*-values for differential gene expression were corrected for multiple testing using the Benjamini and Hochberg algorithm [23], with *P*-value ≤ 0.05 being considered significant. Clustering analysis was conducted on the DEGs detected at 4-h and the 1086 most-highly expressed genes detected at 48-h. Expression values (FPKM) were normalized by the TMM-normalization method (genes were log_2_-transformed and median centered by transcript; Roninson & Oshlack [24]). For DEGs with absolute log_2_ (fold-change) > 1, functions were inferred in a two-stage analysis, as previously described [17].

The RNA-Seq data on which this study was based have been submitted to the NCBI Gene Expression Omnibus (GEO) database under Accession number GSE102664.

## Results

### Microscopic observation of the infection process

**Fig. 1:**
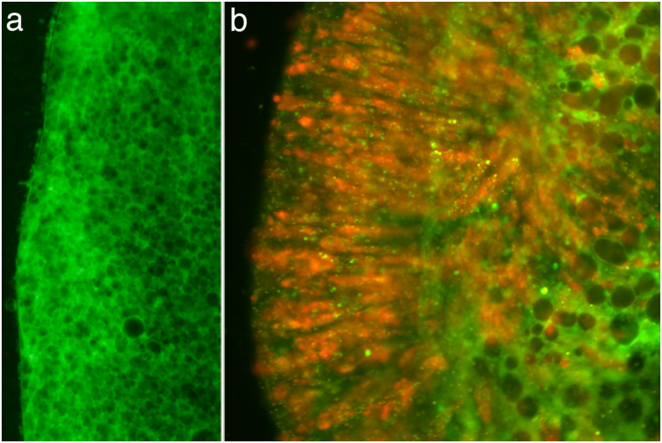
**a** Uninfected *A. digitifera* larva showing the absence of red fluorescence. **b** *Chromera*-infected larva at 4 h post-infection. *Chromera* chloroplasts are responsible for the red fluorescence observed

When *Acropora digitifera* larvae were exposed to the CCMP strain of *Chromera*, intense red fluorescence was observed in the ectoderm of larvae at 4 h post-infection (Fig. 1). As shown in Fig. 1b, the fluorescence is particulate and quite different from the fluorescence of coral RFP [25, 26]. Moreover, no obvious candidates (such as GFP-related or RFP-related proteins) emerged in analyses of early response transcriptomic data (see below). The intense ectodermal fluorescence observed early in the infection process was, however, transient; by 48 h post-infection, only limited fluorescence was visible. Some larvae died during the experiment, but most survived and these contained only small numbers of *Chromera* (if any) after a few days.

### Transcriptome analyses

To understand the nature of the coral–*Chromera* relationship, host transcriptome-wide gene expression changes were determined following exposure of *A. digitifera* larvae to *Chromera* (Fig. 1). At 4, 12, and 48 h post-infection, whole transcriptome expression profiles were compared between *Chromera*-infected and uninfected coral larvae. Sequencing yielded an average of 20 million paired-end (PE) reads per library, and on average 34% of reads were successfully mapped onto the *A. digitifera* transcriptome (Supplementary Tables S1 and S2; Supplementary Fig. S1). Hierarchical clustering of expression data revealed a high level of agreement between the biological replicates, as shown in the pairwise Spearman correlations between samples (Supplementary Figs. S2 and S3). Moreover, multidimensional scaling (based on the 500 genes that best differentiate the samples) resolved the *Chromera*-infected and control samples along the BCV distance 1 (Supplementary Fig. S4).

### Differential gene expression analysis

**Fig. 2:**
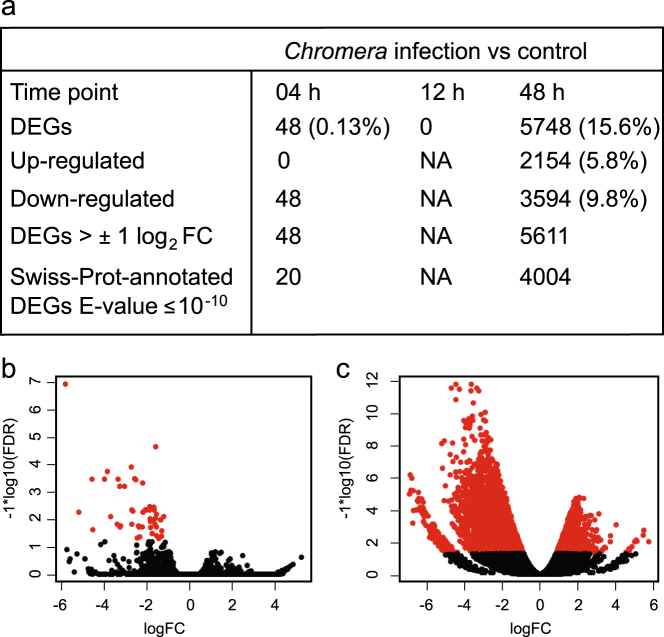
**a** Summary of the differential gene expression profile in *Chromera*-infected larvae compared to controls at 4, 12, and 48 h. An adjusted *P* ≤ 0.05 and E-value cut off ≤10^−10^ were used to filter differentially expressed genes and for BLASTX searches against the Swiss-Prot database. **b**, **c** Volcano plots showing the coral genes differentially expressed at 4 h **b** and 48 h **c** after *Chromera* infection compared to the uninfected control condition. The red dots represent the significantly differentially expressed transcripts at adjusted *P* ≤ 0.05

Distinct transcriptomic responses were detected in the coral host at 4 and 48 h post *Chromera* infection, whereas no significant (using adjusted *P* ≤ 0.05) differential expression was detected at 12 h. At 4 h, relatively few genes (*n* = 48; 0.13% of the coral transcriptome) were downregulated (Fig. 2; Supplementary Fig. S5). However, at 48 h post-infection, the response involved many more genes; 5748 DEGs (about 16% of the coral transcriptome) were differentially expressed, of which 3594 and 2154 genes were downregulated or upregulated, respectively, (Fig. 2; Supplementary Fig. S6). The range of log_2_ (fold-change) and false discovery rates are summarized in Fig. 2. Hierarchical clustering of the 48 DEGs at 4-h and the 1086 most-highly differentially expressed genes at 48-h revealed distinctive expression profiles for *Chromera*-infected and uninfected larvae (Supplementary Figs. S7 and S8).

### The early response profile

Forty-eight *Acropora* genes responded to infection at 4 h, whereas many more (5748) responded at 48 h (Supplementary Fig. S9). Twenty of the 48 had reliable Swiss-Prot annotations (BLASTX E-values ≤ 10^−10^), and amongst these were genes encoding several glycoproteins and members of the immunoglobulin superfamily (Supplementary Table S3). Comparison of the 4 h responses of *A. digitifera* larvae to *Chromera* and *Symbiodinium* [17] revealed only four transcripts in common (Supplementary Fig. S10). Amongst these was adi_EST_assem_1403, which encodes a homolog of the major pancreatic secretory granule membrane glycoprotein GP2. adi_EST_assem_1403 was downregulated to a similar extent in both *Chromera*-infected and *Symbiodinium*-infected larvae (2.72-fold and 2.1-fold, respectively; Supplementary Table S4), suggesting that it may play a central role during coral–algae interactions.

### The late (48 h) response profile—GO and KEGG enrichment

Of those genes differentially expressed at 48 h in response to *Chromera* infection, 4004 had reliable Swiss-Prot annotation (BLASTX E ≤ 10^−10^) (Fig. 2a). Three pathways were enriched amongst the downregulated genes (Benjamini-corrected *P* ≤ 0.05)—*regulation of actin cytoskeleton*, *ECM–receptor interaction*, and *focal adhesion* (Supplementary Tables S5–S8). Mapping against the KEGG database provides more detailed views of the enriched pathways (Supplementary Figs. S11–S13).

Downregulated genes at 48 h showed significant GO enrichment with respect to 27 GO-BP (Biological Process), 43 GO-CC (Cellular Component) and 45 GO-MF (Molecular Function) terms (Supplementary Tables S9–S11). Of the genes downregulated at 48 h, *GTPase regulator activity* was the most overrepresented GO-MF term; also amongst the 122 transcripts in this category were homologs of RAB GTPase activating and binding proteins and members of the TBC1 domain family, all of which play roles in early endosome formation in mammals (Supplementary Table S12, RAB GTPase regulator activity).

Amongst the genes upregulated at 48-h, 14 GO-BP, 63 GO-CC, and 7 GO-MF terms showed significant enrichment (Supplementary Tables S12-S15), the GO-CC term *mitochondrion* and the GO-MF term *structural constituent of ribosome* being the most highly overrepresented terms. Several genes encoding proteins involved in ribosome functions and translation were upregulated (Supplementary Table S16, Ribosome). Many genes encoding mitochondrial ribosomal proteins and components of the electron transport chain including many ATP synthase subunits and proteins of the mitochondrial inner membrane complexes were also upregulated (Supplementary Table S17, Mitochondrion).

### Focus on genes implicated in host–microbe interactions

**Fig. 3:**
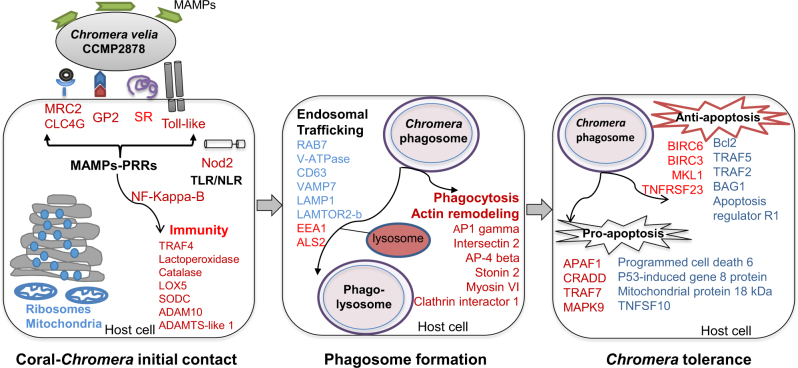
Integrative model of the genes and pathways of larvae of *Acropora digitifera* involved in the interaction with *Chromera.* Upregulated and downregulated genes/functions are in blue and red text, respectively. The initial contact phase (left panel) involves upregulation of ribosomal and mitochondrial functions (based on enrichment of GO terms), and suppression of host immune responses including the downregulation of the pattern recognition receptors (PRRs) that can recognize signature microbial compounds (i.e., microbe-associated molecular patterns or MAMPs). The PRRs that were detected included toll-like receptors (TLRs), a nucleotide-binding oligomerization domain (Nod) protein, scavenger receptors (SRs), lectins, and the complement protein C3. Moreover, the pancreatic secretory granule membrane major glycoprotein GP2, which serves as an uptake receptor for pathogenic bacteria in man, was strongly downregulated. Suppression of PRR–MAMP interactions leads to inactivation of nuclear factor kappa-B (NF-kappa-B), which is a master regulator of immunity. The phagosome formation phase (central panel) involves downregulation of genes involved in phagocytosis and actin remodeling as well as differential expression of genes implicated in endosomal trafficking that enhance the maturation of the phagosome and lysosome fusion (see Fig. 4 for more details about those genes). The *Chromera* tolerance phase (right panel) involves complex changes in the apoptotic network. During this phase, genes likely to have both anti-apoptotic and pro-apoptotic functions were differentially expressed

The GO and KEGG analyses provided a general overview of pathways enriched during the infection process, after which another phase of data analysis was undertaken by grouping the annotated DEGs into categories based on literature searches highlighting functions likely to be involved in host–microbe interactions. During the late response to *Chromera* infection, strong responses were detected in categories of genes associated with immunity, the endocytic pathway, and apoptosis (Fig. 3). The composition of each of these categories of genes is explored in more detail below.

### Suppression of the host immune response at 48 h post-infection

**Fig. 4:**
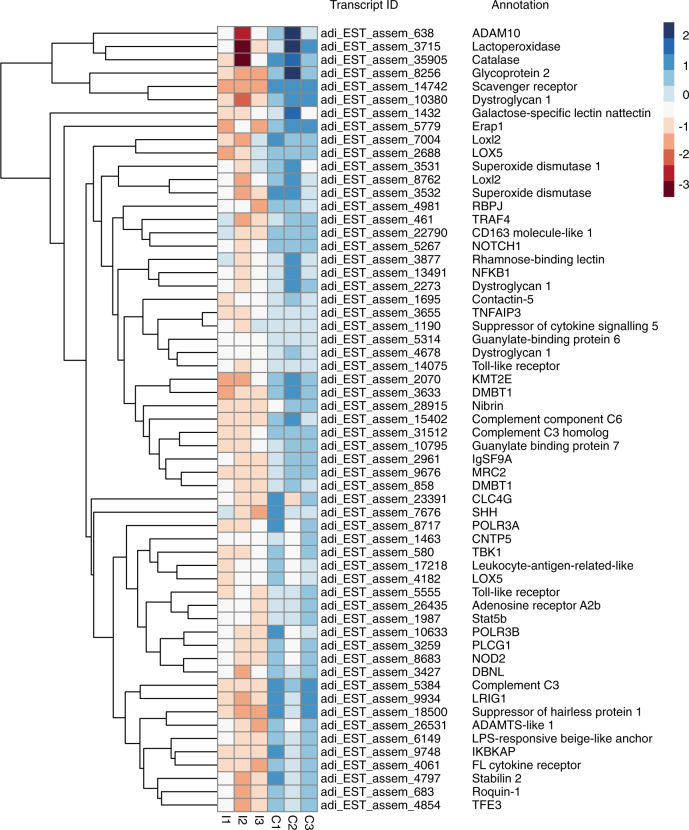
Heat map of genes likely to be involved in immune, inflammatory, stress, and ROS responses in *Chromera*-infected (I1, I2, and I3) and uninfected control larvae (C1, C2, and C3) at 48 h post-infection. The hierarchical clustering shown here was obtained by comparing the expression values (fragments per kilobase of transcript per million; FPKM) for *Chromera*-infected samples against the controls. Expression values were log_2_-transformed and median centered by transcript. The blue-red scale represents the relative expression values

Analyses of the late response of the coral to *Chromera* are consistent with downregulation of host immunity late in the infection process. Sixty *A. digitifera* genes with potential roles in immunity were downregulated at the late time point, including homologs of pattern recognition receptors (PRRs), components of the complement system, genes involved in the TLR/NLR signaling pathways, antimicrobial activities, and genes involved in ROS and inflammatory responses (Fig. 4; Table 1 and Supplementary Table S18).

**Table 1:** Downregulation of *A. digitifera* transcripts likely involved in suppression of the immune response in *Chromera*-infected larvae at 48 h post-infection (FDR ≤ 0.05)

Transcript ID	Best BLASTX Hit	Log FC
adi_EST_assem_5384	Complement C3 (*Mus musculus*; P01027)	−5.12
adi_EST_assem_15402	Complement component C6 precursor (*Homo sapiens*; P13671)	−4.84
adi_EST_assem_31512	Cobra venom factor | Complement C3 homolog (*Naja kaouthia*; Q91132)	−4.1
adi_EST_assem_14742	Scavenger receptor cysteine-rich type 1 protein M160 (*Homo sapiens*; Q9NR16)	−4.47
adi_EST_assem_22790	CD163 molecule-like 1 (*Bos taurus*; P30205)	−1.28
adi_EST_assem_8256	Glycoprotein 2, zymogen granule membrane (*Homo sapiens*; P55259)	−4.18
adi_EST_assem_4678	Dystroglycan 1, dystrophin-associated glycoprotein 1 (*Homo sapiens*; Q14118)	−2.37
adi_EST_assem_10380	Dystroglycan 1, dystrophin-associated glycoprotein 1 (*Canis lupus*; Q9TSZ6)	−2.81
adi_EST_assem_14075	Toll-like receptor (*Acropora digitifera*; aug_v2a.04319)	−1.8
adi_EST_assem_5555	Toll-like receptor (*Acropora digitifera*; aug_v2a.02686)	−1.6
adi_EST_assem_23391	C-type lectin domain family 4-member g (clc4g) (*Acropora digitifera*; aug_v2a.03526)	−3.7
adi_EST_assem_9676	C-type mannose receptor 2 (MRC2) (*Mus musculus*; Q64449)	−1.84
adi_EST_assem_13491	Nuclear factor of kappa light polypeptide gene enhancer in B-cells 1, NF-kappa-B (*Homo sapiens*; P19838)	−1.28
adi_EST_assem_8683	Nucleotide-binding oligomerization domain-containing 2, NOD2 (*Mus musculus*; Q8K3Z0)	−2
adi_EST_assem_461	TNF receptor-associated factor 4 (*Homo sapiens*; Q9BUZ4)	−1.63
adi_EST_assem_3655	Tumor necrosis factor, alpha-induced protein 3 TNFAIP3 (*Homo sapiens*; P21580)	−1.2
adi_EST_assem_9748	Inhibitor of kappa light polypeptide gene enhancer in B-cells, kinase complex-associated protein (*Homo sapiens*; O95163)	−3.5
adi_EST_assem_6149	LPS-responsive beige-like anchor (*Mus musculus*; Q9ESE1)	−2.38
adi_EST_assem_18500	Suppressor of hairless protein 1 (*Xenopus laevis*; Q91880)	−3.19
adi_EST_assem_4061	FL cytokine receptor (*Homo sapiens*; P36888)	−2.37
adi_EST_assem_8717	Polymerase (RNA) III (DNA directed) polypeptide A, 155 kDa (*Homo sapiens*; O14802)	−2.5
adi_EST_assem_10633	polymerase (RNA) III (DNA directed) polypeptide B (*Homo sapiens*; Q9NW08)	−2.67
adi_EST_assem_10795	Guanylate-binding protein 7 (*Homo sapiens*; Q8N8V2)	−2.48
adi_EST_assem_3715	perl_lactoperoxidase (*Bos taurus*; P80025)	−2.54

Columns correspond to the coral transcript ID; best BLASTX hit result, and the log_2_fold-change values

Candidate PRRs whose expression was downregulated included homologs of mannose receptor 2 (MRC2; a c-type lectin) and lectin domain family 4-member G (CLC4G), as well as two scavenger receptors—the cysteine-rich type 1 protein M160 and CD163 molecule-like 1. Expression of three genes encoding homologs of complement system components C3 and C6 precursors was strongly suppressed. The coral homolog of mammalian GP2, which was downregulated at 4 h post-infection (see above), was further downregulated at the 48 h time point, and genes encoding other glycoproteins, including dystroglycan 1, were also downregulated at this time.

Downregulated genes implicated in TLR and NLR signaling pathways included those encoding homologs of two distinct toll-like receptors and NOD2, as well as TRAF4, NF-kappa-B, and TNF alpha-induced protein 3 (TNFAIP3), which are components of the corresponding downstream signaling pathways.

Genes encoding proteins implicated in antimicrobial activity including RNA polymerase III polypeptides A and B (which are key players in sensing and limiting bacterial and DNA viral infection), guanylate-binding protein 7 (which promotes oxidative killing and delivers antimicrobial peptides to phagolysosomes), and lactoperoxidase (which uses hydrogen peroxide and thiocyanate to generate the antimicrobial compound hypothiocyanous acid) were all downregulated. Genes involved in ROS and inflammatory responses including those encoding catalase, superoxide dismutase, and arachidonate 5-lipoxygenase were also downregulated (Fig. 4; Table 1; Supplementary Table S18).

### Modulation of phagocytosis and the endocytic pathway during *Chromera* infection

One characteristic of the response of a coral host to infection with a competent mutualist strain is an apparent stabilization of early endosomes containing the *Symbiodinium* cells [17]. Hence the effect of *Chromera* infection on host phagocytosis and the endocytic pathway is of particular interest.

**Fig. 5:**
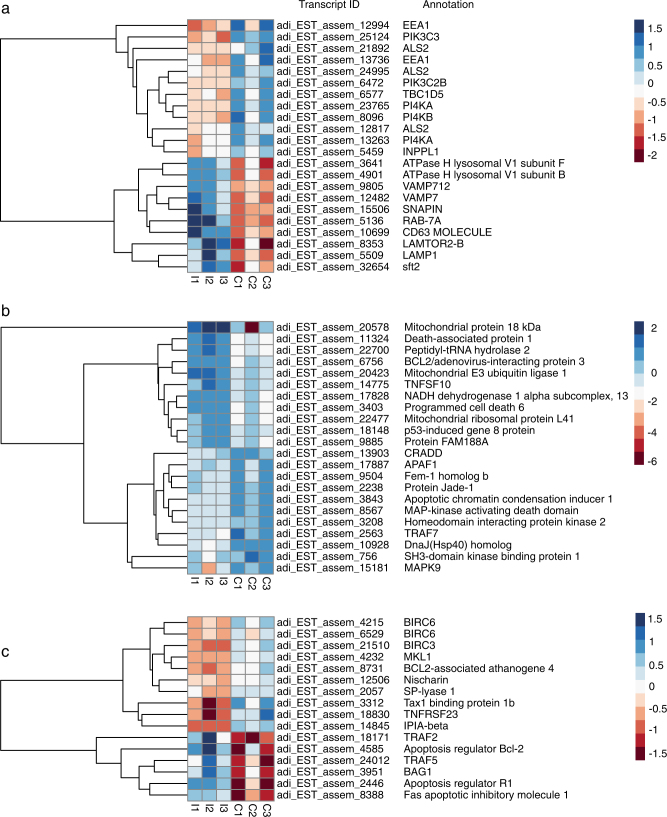
Heat maps of **a** genes likely to be involved in phagosome maturation and lysosomal fusion, **b** genes likely to have pro-apoptotic functions, and **c** genes likely to have anti-apoptotic functions in *Chromera*-infected (I1, I2, I3) and uninfected control larvae (C1, C2, C3) at 48 h post-infection. The hierarchical clustering shown here was obtained by comparing the expression values (fragments per kilobase of transcript per million; FPKM) for *Chromera*-infected samples against the controls. Expression values were log_2_-transformed and median centered by transcript. The blue-red scale represents the relative expression values

**Fig. 6:**
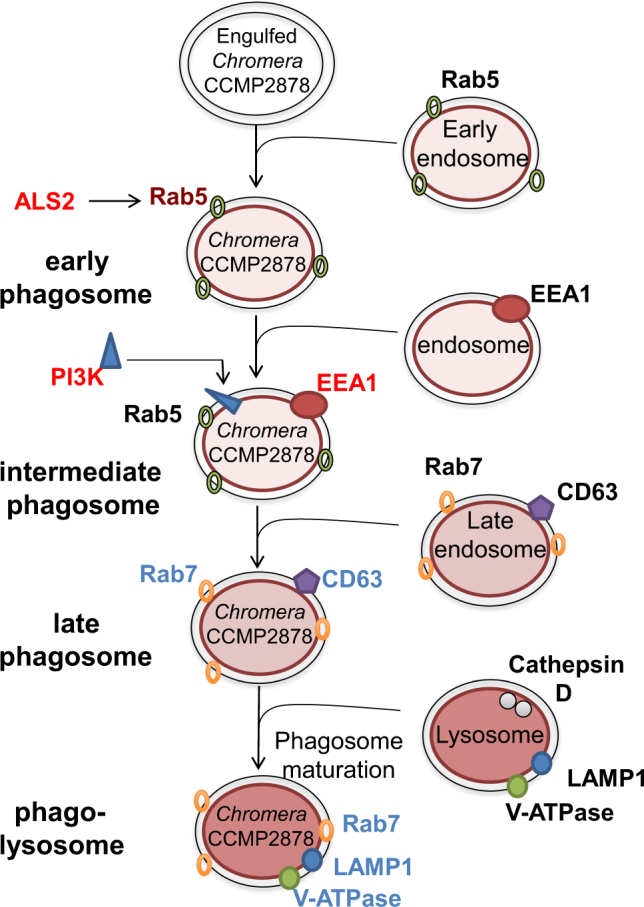
The coral host modulates the endocytic pathway and enhances phagosome maturation and lysosome fusion in response to *Chromera* Upon phagocytosis, the phagosome acquires the GTPase Rab5 via fusion with early endosomes. The Rab5 effector ALS2 was also upregulated. Rab5 acts to recruit phosphatidylinositol-3-kinase (PI3K), which generates phosphatidylinositol-3-phosphate (PI3P) and recruits early endosomal antigen (EEA1) from endosomes. EEA1 is a Rab5 effector protein and its upregulation triggers fusion of the phagosome with a late endosome. During the phagosome maturation process, Rab7 replaces Rab5 and the intermediate phagosome fuses with late endosomal vesicles, acquiring a suite of proteins that includes the proton-ATPase pump (V-ATPase), lysosome-associated membrane glycoprotein 1 (LAMP1), CD63, and lysosomal hydrolases. Vacuoles containing *Chromera* fuse with late endosomes/lysosomes as indicated by the upregulation of genes encoding Rab7, LAMP1, and CD63 (plus late endosomal/ lysosomal adapter, SNAP-associated protein and vesicle-associated membrane protein 7 (VAMP7) “not shown”). The observed upregulation of the lysosomal V-ATPase signals the formation of phagolysosomes, which are typically rich in hydrolytic enzymes and have an extremely low pH, presumably in order to eliminate *Chromera*. Genes in red text are downregulated, while those in blue are upregulated

At the 48 h postinfection time point, 11 genes implicated in phagocytosis were differentially expressed (Supplementary Table S19), all but one of these being downregulated. Twelve genes implicated in the early stages of phagosome maturation were also downregulated, including those encoding the early endosome markers EEA1 (early endosome antigen1) and ALS2 (amyotrophic lateral sclerosis 2) (Fig. 5a; Table 2; Supplementary Table S20); ALS2 is a Rab5 effector protein, which localizes with Rab5 on early endosomal compartments. Conversely, ten genes implicated in the later phase of phagosome maturation were upregulated at the same time point, including those encoding the late phagosome markers Rab7A, CD63, LAMP1 (lysosomal-associated membrane protein 1), TBC1 domain family member 5 (which can displace Rab7A), and LAMTOR2-B, a late endosome/lysosome adapter protein (Table 2; Fig. 6). Differential expression of genes implicated in autophagy and lysosome function was also observed, including upregulation of genes encoding lysosomal thioesterase PPT2-A, iduronidase alpha-l and proteins forming autophagosomal vacuoles and downregulation of lysosomal lipase A and two mannosidases beta A (Supplementary Table S21). In addition, 37 genes encoding proteins involved in endosomal trafficking were differentially expressed including Rab proteins and vacuolar protein sorting proteins (Supplementary Table S22).

**Table 2:** Differential expression of *A. digitifera* transcripts likely involved in early/ late endosome formation and phagosomal maturation in *Chromera*-infected larvae at 48 h post-infection (FDR ≤ 0.05)

	

Down-regulated genes are highlighted in light red whereas up-regulated genes are highlighted in light blue. Columns correspond to the coral transcript ID; best BLASTX hit result, and the log_2_fold-change values

### Complex changes in the apoptotic network during the late response to *Chromera*

A large number of genes whose mammalian homologs are associated with apoptotic responses were differentially expressed at 48 h post-infection (Supplementary Tables S23–S25), but the diversity of roles (likely pro-apoptotic and/or anti-apoptotic functions) complicates interpretation of the biological significance of these data.

Eleven genes predicted to have pro-apoptotic functions were downregulated at 48 h (Fig. 5b; Supplementary Table S23), including genes encoding homologs of the death-domain-containing protein CRADD (which acts as an apoptotic adapter for caspase-2 by recruiting it to the TNFR-1 signaling complex), FEM-1 (which acts as a death receptor-associated protein, thus mediating apoptosis), and the apoptosome scaffold protein APAF1 (Fig. 5b; Supplementary Table S23). Consistent with suppression of apoptosis, genes encoding homologs of six mammalian genes with known anti-apoptotic functions (Fig. 5c; Supplementary Table S24) were upregulated, including a Bcl-2 family member (this *A. digitifera* gene encodes a clear ortholog of the *A. millepora* Mcl1-like protein, for which anti-apoptotic activity has been demonstrated; [27]) and the FAS apoptotic inhibitory molecule.

Conversely, some genes whose products are likely to have anti-apoptotic roles, such as IAP proteins, were downregulated, and others with pro-apoptotic roles upregulated at the same time point (Fig. 5c; Supplementary Tables S23 and S24). Additionally, 28 genes whose products are implicated in modulation of apoptotic pathway activity, including programmed cell death proteins, were differentially expressed (Supplementary Table S25).

## Discussion

*Chromera velia* was initially isolated from a coral and has been considered a possible mutualist, but its mode of entry into coral larvae is very different to that of *Symbiodinium*. Although *Symbiodinium* infection occurs directly into endodermal cells of *Acropora* larvae [28] and does not occur until after the oral pore has opened, *Chromera* is able to infect via the ectoderm. Very rapid entry into host cells is also characteristic of parasitic apicomplexans such as *Toxoplasma* and *Plasmodium*, where the active process can be effected in 20–30 s [29]. In contrast to these other apicomplexans, little is known about the infective stage of *Chromera*. However, Oborník et al. [30] have described three distinct morphological stages (coccoid, cystic, and flagellated), of which the flagellated stage seems the best candidate for infection. The flagellated stage develops a unique structure known as the “chromerosome”, which Oborník et al. [30] hypothesized might be involved in penetration of coral cells.

Massive invasion of coral planulae by *Chromera* (i.e., as shown in Fig. 1b) has not previously been documented despite similar experiments having been conducted with *Chromera* at the same location and with the same host species. However, the initial sampling point used by Cumbo et al. [5] was one day after exposure, by which time fluorescence was greatly reduced in the present study. Another possibility is that the coral response to the CCMP strain of *Chromera* (used here) differs fundamentally from the response to stains derived from tropical corals (as used in the [5] study).

The timing and scale of the transcriptomic response of *A. digitifera* larvae to *Chromera* differed markedly from that to a competent *Symbiodinium* strain [17]. In the latter case, significant changes were detected only at the 4 h time point (although note that a recent study documented subtle transcriptomic changes at 6 d post-infection [31] and involved <3% of transcripts, whereas in the case of *Chromera* infection, the response occurred predominantly on a longer timescale, but involved many more host genes (>16% of transcripts). While no strictly comparable data are yet available, the coral response to *Chromera* was more similar to the response to a non-competent strain of *Symbiodinium* than to a competent strain in that the former provoked more extensive responses on longer time scale [16]. Direct comparison of the data presented here with the literature is complicated by differences in timing—in the present case 4, 12, and 48 h post-infection, whereas Voolstra et al. [16] used 30 min and 6 d sampling times. Some of the same genes were differentially expressed at the late time point in both cases, but the direction of change sometimes differed between the two studies (Supplementary Table S26). Genes implicated in signal transduction, transport, cell adhesion, and recognition were downregulated in response to *Chromera,* but were upregulated in response to non-competent *Symbiodinium*, implying that while the coral reacts by attempting to limit interaction with *Chromera*, in the case of non-competent *Symbiodinium* a negotiation phase may occur, involving (for example) upregulation of c-type lectin.

Despite the distinct responses of coral larvae to *Chromera* and *Symbiodinium* [17], one common characteristic was downregulation of the gene encoding the coral homolog of pancreatic secretory granule membrane major glycoprotein GP2 at 4 h post-infection. In the case of infection with a competent *Symbiodinium* strain, GP2 expression had returned to baseline levels by 12 h post-infection [17], whereas the gene was further downregulated 48 h after *Chromera* infection. In man, GP2 is specifically expressed on membranous cells (M cells) where it acts as a bacterial uptake receptor and is involved in initiating an innate immune response [32–34]. While the role of coral GP2 protein is unknown, its downregulation in response to the presence of potential symbionts suggests that it might function in host–algal recognition in an analogous manner to its mammalian homologs.

### The coral late (48 h) response to *Chromera* infection

In contrast to the 4 h response, the 48 h response of the coral host to *Chromera* was extensive and complex. As indicated above, the GO molecular function *GTPase regulator activity* was highly overrepresented amongst downregulated genes, indicating modulation of the host endocytic pathway, which plays critical roles not only in host–pathogen interactions, such as the mammalian response to *Mycobacterium tuberculosis* [35], but also in the establishment of symbiosis in corals [17] and sea anemones [36, 37]. Amongst upregulated genes, the GO terms *mitochondrion* and *structural constituent of ribosome* (ribosomal proteins) were highly overrepresented, implying that *Chromera* infection resulted in the activation of protein synthesis and metabolism. While responses like this have been reported in the sea fan *Gorgonia ventalina* after exposure to the parasite *Aplanochytrium* [38] and in other invertebrates [39, 40], these results are in complete contrast to the responses of coral larvae to a competent strain of *Symbiodinium*, where protein synthesis and metabolism were temporarily suppressed [17].

### Modulation of phagosome maturation in the coral host in order to eliminate *Chromera*

Phagocytosis is well understood in vertebrate phagocytes, such as macrophages, which engulf and destroy non-self cells or microbial invaders. Immediately after phagocytosis, a specific marker, the small GTPase Rab5, is recruited to the phagosome resulting in fusion with an early endosome [41]. Rab5 recruits phosphatidylinositol-3-phosphate (PI3K), which generates phosphoinositol 3-phosphate PI(3)P, which in turn recruits early endosome antigen1 (EEA1) from the endosome. EEA1 is a Rab5 effector protein that triggers fusion of a phagosome with a late endosome [35]. The phago-lysosome is a microbicidal environment that includes antimicrobial peptides and hydrolases. Some pathogenic microbes have evolved strategies enabling them to survive inside host phagocytes (see below), whereas *Leishmania mexicana* and *Coxiella burnetii* are well adapted to the highly acidic environment of phagolysosomes and *Trypanosoma cruzi* has evolved mechanisms enabling escape from the phagosome [35, 42, 43].

Host endocytic pathway gene expression in response to *Chromera* infection is very different to that observed with a competent strain of *Symbiodinium* [17]. During *Chromera* infection, many genes involved in phagocytosis (e.g., those encoding PRRs) were downregulated, resulting in reduced recognition of invading *Chromera* and suppression of host immune responses. Downregulation of genes involved in actin remodeling and enrichment of the KEGG pathways *regulation of actin cytoskeleton* and *focal adhesion* amongst downregulated genes suggest that actin remodeling is required at the site of phagocytosis in order to prevent the formation and extension of pseudopods. Genes encoding a range of early endosome markers (Rab5 effector proteins (EAA1 and ALS2)) and phosphoinositide-3 and 4-kinases (PI3K and PI4K) were downregulated during *Chromera* infection (Table 2). In addition, genes involved in phagosome maturation were upregulated, including markers of the late endosome (Rab7a, LAMP1, VAMPs, and CD63) and of the vesicle fusion process (late endosomal / lysosomal adapter, SNAP-associated protein and VAMP7). Genes encoding two subunits of the lysosomal proton-ATPase were also upregulated, and others encoding vacuolar sorting proteins, TBC1 domain members, other Rab proteins, and lysosomal hydrolases had altered expression compared to the control, indicating the dynamic nature of the endosomal pathway. In contrast to the *Chromera* data, during infection of coral larvae with a competent strain of *Symbiodinium*, early endosome markers were upregulated, whereas molecular data for both the coral and a symbiotic sea anemone are consistent with the symbiosome being essentially an arrested early phagosome [17, 36, 37], these results indicate that the coral host responds to *Chromera* by upregulating phagosome maturation, presumably to promote fusion with late endosomes and/or lysosomes in order to eliminate *Chromera*.

In addition to its role in the establishment of a stable cnidarian–*Symbiodinium* interaction, the endocytic pathway is manipulated in various ways by bacterial pathogens and parasites to gain entry to the cell [44]. For example, like *Symbiodinium* [17], pathogenic strains of *M. tuberculosis* [35] arrest phagosome maturation at an early stage, retaining Rab5, but inhibiting both Rab7 acquisition [45] and recruitment of the H^+^-ATPase to the phagosomal membrane [46]. Non-pathogenic strains of *M. tuberculosis* do not have the ability to arrest phagosome maturation [35].

### Suppression of the host immune response and apoptosis after *Chromera* infection

Cnidarian genomes encode many of the innate immune components known from vertebrates, including those involved in pattern recognition, signaling cascades, and effector responses [9, 47]. At 48 h post-infection, expression of a range of PRRs and other genes, including those encoding C-type lectins, scavenger receptors, complement components, TLRs, NLRs, and GP2 was strongly suppressed, implying that host immunity may be downregulated or compromised in response to *Chromera* infection. Downregulation of some PRRs could reflect the host attempting to limit interactions with non-beneficial organisms; for example, complement C3 and the C-type lectin, mannose receptor 2 (MRC2), have been implicated in symbiont recognition. Kvennefors et al. [48] localized a C3 homolog near the resident symbionts in the coral *A. millepora*, suggesting that C3 might act as an opsonin with respect to *Symbiodinium* and play a role in host-symbiont communication. MRC2 was highly upregulated in *A. digitifera* larvae infected with a competent *Symbiodinium* strain [17], suggesting a function in host-symbiont recognition during establishment of coral–algal symbioses. Thus, downregulating the expression of these genes might reflect the host attempting to limit uptake of *Chromera*. Downregulation of a scavenger receptor might also reflect a response of this type, as a scavenger receptor has been implicated in communication between the symbiotic sea anemone *Anthopleura elegantissima* and its resident *Symbiodinium* [49].

On the other hand, some of the observed differences in immune gene expression may reflect suppression of host immunity by *Chromera*. Downregulation of genes encoding antimicrobial activities (such as guanylate-binding protein 7 and lactoperoxidase) in response to *Chromera* infection clearly suggests the ability of the invading *Chromera* to deactivate the host-killing mechanisms, resulting in survival inside the host. Scavenger receptors have been implicated in the recognition of pathogenic *Mycobacterium* [50], and TLRs and NLRs are involved in sensing the malaria parasite *Plasmodium* spp. in different vertebrate hosts [51]. Antimicrobial activities are often induced in response to TLR activation [52]. Pathogenic strains of *Mycobacterium* use the mannose-capped lipoglycan, mannosylated lipoarabinomannan, in order to inhibit TLR signaling and impair T-cell activation.

However, the observed suppression of the immune response by *Chromera* contrasts with the response of the Caribbean Sea fan, *Gorgonia ventalina*, to infection with the parasite *Aplanochytrium*, where genes encoding PRRs and likely antimicrobial activities (including guanylate-binding protein) were upregulated in response to the parasite [38]. The *Chromera* result also stands in contrast with data from corals infected with white syndrome and white band diseases [11, 53]. Moreover, activation of coral immune responses has been reported in response to viral and bacterial mimics [15] and challenge with pathogenic bacteria [54].

The observed downregulation of a suite of pro-apoptotic genes (those encoding TNF receptors and corresponding pathway components, APAF1 and cytochrome c-mediated cell death pathway genes) and upregulation of anti-apoptotic genes (Bcl-2, TRAF2, and TRAF5), implies that apoptosis is inhibited in coral larvae following *Chromera* infection. It is unclear, however, whether these changes reflect host adaptation to tolerate *Chromera* infection, or manipulation of the coral response by *Chromera*. Pathogenic microbes including *M. tuberculosis* have adapted to their host cells by developing anti-apoptosis mechanisms that may reduce the effects of apoptosis-inducing components [55], and a similar strategy appears to be in place in *Helicobacter pylori* [56].

### The relationship between *Chromera* and corals—parasite or innocent bystander?

The association between apicomplexans and corals has been known for some time (e.g., [1, 57]), but the description of *Chromera* in 2008 [2] and the availability of abundant sequence data from corals have spurred interest in their apicomplexan associates. Kirk et al. [3] used PCR to investigate the presence and seasonal abundance of apicomplexans in four coral species in Florida and the Bahamas for periods of up to nine years and found the associations to be ubiquitous, although seasonably variable. However, rather than *Chromera*, the most abundant apicomplexan was an undescribed organism presently known only as “genotype N”. In an effort to clarify the diversity of apicomplexans associated with coral and coral reefs, Janouškovec et al. [4] examined prokaryotic sequence surveys for DNA from eukaryotic plastids. From these surveys they recognized eight groups of apicomplexan-related lineages, only two of which—*Chromera* and *Vitrella*—have been described and characterized. They also suggested that *Chromera* was associated with the surface of corals rather than with coral tissue, whereas the presently uncharacterized apicomplexan ARL-V was associated specifically with coral tissue rather than with the surface of the coral. That ARL-V was closely associated with the coral while both *Chromera* and *Vitrella* were more-closely associated with macroalgae growing near the reef was later confirmed by analysis of data from a fine-scale 16S profiling survey along transects that included a reef and associated macroalgae [7, 58]. On the basis of this survey, the authors state “This suggests, contrary to common belief, that *Vitrella* and *Chromera* may not be obligate coral symbionts, and possibly interact indirectly with coral.”—a conclusion with which the transcriptomic data reported here are consistent. Also, it should be stressed that the absolute abundance of *Chromera* sequences in these surveys is very low, again suggesting that the association with corals may be somewhat accidental, and is certainly not obligate. However, abundance is not necessarily correlated with functional importance in coral-associated microbes [59].

## Conclusion

Despite the level of interest in *Chromera*, very little is known about the relationship of this novel alga with corals. In the present case, the response of *A. digitifera* larvae to the CCMP strain of *Chromera* differed markedly from that to a competent strain of the coral mutualist *Symbiodinium*, instead being more similar to the response to non-competent (“incompetent”) strains of *Symbiodinium* [16] in extent and timescale (although note that strictly comparable data are not available). Although infection with a competent *Symbiodinium* strain resulted in arrest of the host endocytic pathway at the early phagosome stage, in the case of *Chromera* infection, phagosome maturation appeared to be stimulated.

Together with the observed modulation of both the immune and apoptotic networks, these data point to a hostile response to *Chromera* by coral larvae, and macroscopic observation suggests that *Chromera* cells are eventually largely cleared despite their initial efficient uptake (Fig. 1); one apparent paradox is that clearing appears to occur more rapidly than can be accounted for by the transcriptional responses documented here. The most likely explanations are that either gene expression changes occurring between the 12 and 48 h sampling time points, or post-transcriptional processes (such as the activation of apoptotic pathways)—or both of these—are responsible for *Chromera* clearance. Regardless, the abundance of *Chromera* associated with corals in nature appears to be low—our own attempts to survey their distribution support this—presumably as a consequence of hostile response of the coral to *Chromera* infection.

While the work described here, and the sequence survey literature, imply that *Chromera* is not a coral mutualist, the extent to which the observations made here with the CCMP (Sydney Harbor) strain of *Chromera* hold more generally is not clear. Anecdotally, *Chromera* is a diverse genus and, in the case of *Symbiodinium*, high genetic diversity plays out in a spectrum of host responses (for example, [16]). A better understanding of the nature of the coral–*Chromera* interaction will require estimation of the genetic diversity of *Chromera*.

## Electronic supplementary material

Supplementary file

Supplementary file

## References

[1] Toller W, Rowan R, Knowlton N (2002). Genetic evidence for a protozoan (phylum Apicomplexa) associated with corals of the *Montastraea annularis* species complex. Coral Reefs.

[2] Moore RB, Oborník M, Janouškovec J, Chrudimský T, Vancová M, Green DH (2008). A photosynthetic alveolate closely related to apicomplexan parasites. Nature.

[3] Kirk NL, Thornhill DJ, Kemp DW, Fitt WK, Santos SR (2013). Ubiquitous associations and a peak fall prevalence between apicomplexan symbionts and reef corals in Florida and the Bahamas. Coral Reefs.

[4] Janouškovec J, Horak A, Barott KL, Rohwer FL, Keeling PJ (2012). Global analysis of plastid diversity reveals apicomplexan-related lineages in coral reefs. Curr Biol.

[5] Cumbo VR, Baird AH, Moore RB, Negri AP, Neilan BA, Salih A (2013). *Chromera velia* is endosymbiotic in larvae of the reef corals *Acropora digitifera* and *A. tenuis*. Protist..

[6] Visser P, Bintoudi E, Boschker E, Frade P, van Bleijswijk J, Matthijs H et al. Newly discovered coral endosymbiont *Chromera* is more thermotolerant than *Symbiodinium.*In: 12th Int Coral Reef Symp Abstracts. Queensland: James Cook University; 2012, p. 198.

[7] Janouškovec J, Horák A, Barott KL, Rohwer FL, Keeling PJ (2013). Environmental distribution of coral-associated relatives of apicomplexan parasites. ISME J..

[8] Jenner RG, Young RA (2005). Insights into host responses against pathogens from transcriptional profiling. Nat Rev Microbiol.

[9] Miller DJ, Hemmrich G, Ball EE, Hayward DC, Khalturin K, Funayama N (2007). The innate immune repertoire in Cnidaria-ancestral complexity and stochastic gene loss. Genome Biol.

[10] Anderson DA, Walz ME, Weil E, Tonellato P, Smith MC (2016). RNA-Seq of the Caribbean reef-building coral *Orbicella faveolata* (Scleractinia-Merulinidae) under bleaching and disease stress expands models of coral innate immunity. PeerJ.

[11] Libro S, Kaluziak ST, Vollmer SV (2013). RNA-seq profiles of immune related genes in the staghorn coral *Acropora cervicornis* infected with White Band disease. PLoS ONE.

[12] Ocampo ID, Zárate-Potes A, Pizarro V, Rojas CA, Vera NE, Cadavid LF (2015). The immunotranscriptome of the Caribbean reef-building coral *Pseudodiploria strigosa*. Immunogenetics.

[13] Pinzón JH, Kamel B, Burge CA, Harvell CD, Medina M, Weil E (2015). Whole transcriptome analysis reveals changes in expression of immune-related genes during and after bleaching in a reef-building coral. Roy Soc Open Sci.

[14] Vidal-Dupiol J, Ladriere O, Meistertzheom AL, Foure L, Adjeroud M, Mitta G (2011). Physiological responses of the scleractinian coral *Pocillopora damicornis* to bacterial stress from *Vibrio coralliilyticus*. J Exp Biol.

[15] Weiss Y, Forêt S, Hayward DC, Ainsworth T, King R, Ball EE (2013). The acute transcriptional response of the coral *Acropora millepora* to immune challenge: expression of GiMAP/IAN genes links the innate immune responses of corals with those of mammals and plants. BMC Genomics.

[16] Voolstra CR, Schwarz JA, Schnetzer J, Sunagawa S, Desalvo MK, Szmant AM (2009). The host transcriptome remains unaltered during the establishment of coral–algal symbioses. Mol Ecol.

[17] Mohamed AR, Cumbo V, Harii S, Shinzato C, Chan CX, Ragan MA (2016). The transcriptomic response of the coral *Acropora digitifera* to a competent *Symbiodinium* strain: the symbiosome as an arrested early phagosome. Mol Ecol.

[18] Shinzato C, Shoguchi E, Kawashima T, Hamada M, Hisata K, Tanaka M (2011). Using the *Acropora digitifera* genome to understand coral responses to environmental change. Nature.

[19] Guillard RRL, Ryther JH (1962). Studies of marine planktonic diatoms. I. *Cyclotella nana* Hustedt and *Detonula confervacea Cleve*. Can J Microbiol..

[20] Langmead B, Trapnell C, Pop M, Salzberg SL (2009). Ultrafast and memory-efficient alignment of short DNA sequences to the human genome. Genome Biol..

[21] Li B, Dewey CN (2011). RSEM: accurate transcript quantification from RNA-Seq data with or without a reference genome. BMC Bioinformatics.

[22] Robinson MD, McCarthy DJ, Smyth GK (2010). edgeR: a Bioconductor package for differential expression analysis of digital gene expression data. Bioinformatics.

[23] Benjamini Y & Hochberg Y. Controlling the false discovery rate: a practical and powerful approach to multiple testing. J R Stat Soc Series B Stat Methodol. 1995; 57:289–300.

[24] Roninson M, Oshlack A (2010). A scaling normalization method for differential expression analysis of RNA-seq data. Genome Biol.

[25] Strader ME, Aglyamova GV, Matz MV (2016). Red fluorescence in coral larvae is associated with a diapause‐like state. Mol Ecol.

[26] Beltran-Ramirez V. Molecular aspects of the fluorescent protein homologues in *Acropora millepora*. PhD thesis, James Cook University, QLD, 2010.

[27] Moya A, Sakamaki K, Mason BM, Huisman L, Forêt S, Weiss Y (2016). Functional conservation of the apoptotic machinery from coral to man: the diverse and complex Bcl-2 and caspase repertoires of *Acropora millepora*. BMC Genomics.

[28] van Oppen M (2001). *In vitro* establishment of symbiosis in *Acropora millepora* planulae. Coral Reefs.

[29] Plattner F, Soldati-Favre D (2008). Hijacking of host cellular functions by the Apicomplexa. Annu Rev Microbiol.

[30] Oborník M, Vancová M, Lai DH, Janouškovec J, Keeling PJ, Lukeš J (2011). Morphology and ultrastructure of multiple life cycle stages of the photosynthetic relative of apicomplexa. Chrome velia Protist.

[31] Wolfowicz I, Baumgarten S, Voss PA, Hambleton EA, Voolstra CR, Hatta M (2016). *Aiptasia* sp. larvae as a model to reveal mechanisms of symbiont selection in cnidarians. Sci Rep.

[32] Hase K, Kawano K, Nochi T, Pontes GS, Fukuda S, Ebisawa M (2009). Uptake through glycoprotein 2 of FimH + bacteria by M cells initiates mucosal immune response. Nature.

[33] Ohno H, Hase K (2010). Glycoprotein 2 (GP2): grabbing the FimH bacteria into M cells for mucosal immunity. Gut Microbes.

[34] Yu S, Lowe AW (2009). The pancreatic zymogen granule membrane protein, GP2, binds *Escherichia coli* Type 1 fimbriae. BMC Gastroenterol.

[35] Koul A, Herget T, Klebl B, Ullrich A (2004). Interplay between mycobacteria and host signalling pathways. Nat Rev Microbiol.

[36] Chen MC, Cheng YM, Hong MC, Fang LS (2004). Molecular cloning of Rab5 (ApRab5) in *Aiptasia pulchella* and its retention in phagosomes harboring live zooxanthellae. Biochem Biophys Res Commun.

[37] Chen MC, Cheng YM, Sung PJ, Kuo CE, Fang LS (2003). Molecular identification of Rab7 (ApRab7) in *Aiptasia pulchella* and its exclusion from phagosomes harboring zooxanthellae. Biochem Biophys Res Commun.

[38] Burge CA, Mouchka ME, Harvell CD, Roberts S (2013). Immune response of the Caribbean sea fan, *Gorgonia ventalina*, exposed to an *Aplanochytrium* parasite as revealed by transcriptome sequencing. Front Physiol.

[39] Gestal C, Costa M, Figueras A, Novoa B (2007). Analysis of differentially expressed genes in response to bacterial stimulation in hemocytes of the carpet-shell clam *Ruditapes decussatus*: identification of new antimicrobial peptides. Gene.

[40] Travers MA, Meistertzheim AL, Cardinaud M, Friedman CS, Huchette S, Moraga D (2010). Gene expression patterns of abalone, *Haliotis tuberculata*, during successive infections by the pathogen *Vibrio harveyi*. J Invertebr Pathol.

[41] Vieira OV, Botelho RJ, Grinstein S (2002). Phagosome maturation: aging gracefully. Biochem J..

[42] Flannagan RS, Cosio G, Grinstein S (2009). Antimicrobial mechanisms of phagocytes to bacterial evasion strategies. Nat Rev Microbiol.

[43] Sacks D, Sher (2002). Evasion of innate immunity by parasitic protozoa. Nat Immunol.

[44] Gruenberg J, van der Goot FG (2006). Mechanisms of pathogen entry through the endosomal compartments. Nat Rev Mol Cell Biol.

[45] Sun J, Deghmane AE, Soualhine H, Hong T, Bucci C, Solodkin A (2007). *Mycobacterium bovis* BCG disrupts the interaction of Rab7 with RILP contributing to inhibition of phagosome maturation. Jour Leukoc Biol..

[46] Sturgill-Koszycki S, Schlesinger PH, Chakraborty P, Haddix PL, Collins HL, Fok AK (1994). Lack of acidification in *Mycobacterium* phagosomes produced by exclusion of the vesicular proton-ATPase. Science..

[47] Augustin R, Bosh TC (2010). Cnidarian immunity: a tale of two barriers. Adv Exp Med Biol.

[48] Kvennefors ECE, Leggat W, Kerr CC, Ainsworth TD, Hoegh-Guldberg O, Barnes AC (2010). Analysis of evolutionarily conserved innate immune components in coral links immunity and symbiosis. Dev Comp Immunol.

[49] Rodriguez-Lanetty M, Phillips WS, Weis VM (2006). Transcriptome analysis of a cnidarian-dinoflagellate mutualism reveals complex modulation of host gene expression. BMC Genomics..

[50] Killick KE, Ní Cheallaigh C, O’Farrelly C, Hokamp K, MacHugh DE, Harris J (2013). Receptor mediated recognition of mycobacterial pathogens. Cell Microbiol..

[51] Gazzinelli RT, Kalantari P, Fitzgerald KA, Golenbock DT (2014). Innate sensing of malaria parasites. Nat Rev Immunol..

[52] Davies D, Meade KG, Herath S, Eckersall PD, Gonzalez D, White JO (2008). Toll-like receptor and antimicrobial peptide expression in the bovine endometrium. Reprod Biol Endocrinol..

[53] Wright RM, Aglyamova GV, Meyer E, Matz MV (2015). Gene expression associated with white syndromes in a reef building coral, *Acropora hyacinthus*. BMC Genomics..

[54] Brown T, Bourne D, Rodriguez-Lanetty M (2013). Transcriptional activation of c3 and hsp70 as part of the immune response of *Acropora millepora* to bacterial challenges. Plos One..

[55] Briken V, Miller JL (2008). Living on the edge: inhibition of host cell apoptosis by *Mycobacterium tuberculosis*. Future Microbiol..

[56] Mimuro H, Suzuki T, Nagai S, Rieder G, Suzuki M, Nagai T (2007). *Helicobacter pylori dampens gut epithelial* self-renewal by inhibiting apoptosis, a bacterial strategy to enhance colonization of the stomach. Cell Host Microbe..

[57] Peters EC (1984). A survey of cellular reactions to environmental stress and disease in Caribbean scleractinian corals. Helgol Mar Res..

[58] Barott KL, Rodriguez-Brito B, Janouškovec J, Marhaver K, Smith JE, Keeling P (2011). Microbial diversity associated with four functional groups of benthic reef algae and the reef-building coral *Montastraea annularis*. Environ Microbiol..

[59] Ainsworth T, Krause L, Bridge T, Torda G, Raina JB, Zakrzewski M (2015). The coral core microbiome identifies rare bacterial taxa as ubiquitous endosymbionts. ISME J..

