# Supplementary material for: Deciphering the nature of the coral–Chromera association
Source: ISME J. 2018 Jan 10;12(3):776–90. doi: 10.1038/s41396-017-0005-9 (PMC5864212; doi:10.1038/s41396-017-0005-9)
Supplement: Supplementary file 1 — Supplementary file [file 41396_2017_5_MOESM1_ESM.docx]

**Supplementary Information**

**Deciphering the nature of the coral-*Chromera* association**

Amin R Mohamed^1,2,3,4^, Vivian Cumbo^1,5^, Saki Harii^6^, Chuya Shinzato^7^, Cheong Xin Chan^8^, Mark A Ragan^8^, Nori Satoh^7^, Eldon E Ball^9^, David J Miller^1,2^

Affiliations:

^1^ARC Centre of Excellence for Coral Reef Studies, James Cook University, Townsville 4811, Queensland, Australia

^2^Comparative Genomics Centre and Department of Molecular and Cell Biology, James Cook University, Townsville 4811, Queensland, Australia

^3^Zoology Department, Faculty of Science, Benha University, Benha 13518, Egypt

^4^AIMS@JCU, Australian Institute of Marine Science, Department of Molecular and Cell Biology, James Cook University, Townsville 4811, Queensland, Australia

^5^Department of Biological Sciences, Macquarie University, Sydney, NSW 2109, Australia

^6^Sesoko Station, Tropical Biosphere Research Center, University of the Ryukyus, 3422 Sesoko, Motobu Okinawa 905-0227, Japan

^7^Marine Genomics Unit, Okinawa Institute of Science and Technology Promotion Corporation, Onna, Okinawa 904-0412, Japan

^8^Institute for Molecular Bioscience, The University of Queensland, Brisbane, QLD 4072, Australia

^9^Division of Ecology and Evolution, Research School of Biology, Australian National University, Acton, ACT 2601, Australia

*Present address: Atmosphere and Ocean Research Institute, The University of Tokyo, 5-1-5, Kashiwanoha, Kashiwa-shi, Chiba 277-8564 Japan

**Supplementary** **Methods**

***Mapping Illumina reads***

Reads were mapped onto the *Acropora digitifera* transcriptome (Shinzato *et al.*, 2011) (<http://marinegenomics.oist.jp/genomes/>) using the BOWTIE mapping software version 0.12.7 (Langmead *et al.*, 2009) (<http://bowtie-bio.sourceforge.net/index.shtml>). The alignment (bam) files were fed to RSEM software version 1.1.17 (<http://deweylab.biostat.wisc.edu/rsem/>) to generate the abundance estimation for each sample (Li and Dewey, 2011). The read alignments and the reference sequences were visualized as a sanity check using the Integrated Genomics Viewer (IGV) software version 2.3.34 (Thorvaldsdottir *et al.*, 2013) (<http://www.broadinstitute.org/igv/>). Also percentages of mapped reads were obtained by using the samtools flagstat command.

***Differential Gene Expression analysis***

The R packages edgeR (Robinson *et al.*, 2010) and DESeq (Anders and Huber, 2010) were compared for the differential gene expression analysis. EdgeR showed higher numbers of DEGs at adjusted *P* ≤ 0.05 than DESeq (5748 DEGs versus 2638 DEGs, respectively) hence edgeR results were used.

EdgeR measures gene expression (transcript counts) modeled with a Negative Binomial (NB) distribution and determines differential expression using empirical Bayesian estimation and exact tests based on the NB model.

The plot_MA_and_Volcano function in R was also used to generate MA plots and volcano plots of the DEGs at FDR ≤ 0.05. To study expression patterns of genes across samples, raw counts were first normalized using the TMM normalization in edgeR to scale the FPKM (expression) values provided by the RSEM software across all samples. The R package heatmap3 (<https://cran.r-project.org/web/packages/heatmap3/index.html>) was used to generate sample Spearman correlation and gene clustering heat maps.

Heat maps of specific categories of the DEGs likely involved in host-microbe interactions were generated using the R package pheatmap (<https://cran.r-project.org/web/packages/pheatmap/index.html>).

***Gene ontology (GO) enrichment analysis using DAVID***

The UniProt accessions were used as identifiers for enrichment analysis of Gene Ontology (GO) annotations using DAVID (Huang da *et al.*, 2009). The *A. digitifera* transcriptome was used as query in BLASTx searches against the Swiss-Prot database. Transcripts with GO terms annotated based on their Swiss-Prot hits were used as the background set for enrichment analysis. DAVID uses Fisher’s exact test to ascertain statistically significant GO enrichment amongst differentially expressed transcripts relative to the background.

***Supplementary*** ***Results***

**GO enrichment in the late response to *Chromera***

Down-regulated genes at 48 h showed significant GO enrichment to 27 terms related to Biological Process including regulation of small GTPase mediated signal transduction, transcription, and RNA metabolic process (Supplementary Table S9); 43 terms related to Cellular Component including cytoskeleton (Supplementary Table S10); and 45 terms related to molecular function including GTPase regulator activity (Table S11). The molecular function term ‘GTPase regulator activity’ was the most highly over-represented category amongst down-regulated genes. Amongst the 122 *A. digitifera* genes that are annotated with this term are RAB GTPase activating and binding proteins and members of TBC1 domain family that play important roles during early endosome formation (Supplementary Table S12).

On the other hand, up-regulated genes at 48 h showed significant enrichment to 14 terms related to Biological Process including translation and electron transport chain (Supplementary Table S13); 36 terms related to Cellular Component including mitochondrion and ribosome (Supplementary Table S14); and 7 terms related to Molecular Function including structural constituent of ribosome (Supplementary Table S15). The cellular component term *mitochondrion* and the molecular function term *structural constituent of ribosome* were the most highly over-represented amongst up-regulated genes. Several genes encoding proteins involved in ribosome functions and translation including ribosomal proteins were also up-regulated (Ribosome- Supplementary Table S16). Many genes encoding mitochondrial ribosomal proteins and components of the electron transport chain including many ATP synthase subunits and proteins of the mitochondrial inner membrane complexes were up-regulated (Mitochondrion**-**Supplementary Table S17).

**Genes involved in** **endosomal trafficking were affected during the late response to *Chromera***

Rab proteins required for protein transport from the endoplasmic reticulum to the Golgi complex, transport between the plasma membrane and early endosomes, and transport between the endosomes and the trans-Golgi network including Rab18B, Rab9A, Rab36, Rab10, Rab28, Rab4-like, Rab2, Rab3, as well as the protein transport protein SFT2, were up-regulated with fold change range from 1.13 to 2.04. Moreover one gene encoding a SNAP-associated protein that is involved in SNARE-mediated membrane fusion was up-regulated with 1.37-fold increase. However, genes encoding Rab GTPase activating protein 1-like, Rab11 family interacting protein 3, Rab3 GTPase activating protein subunits 1, 2 and Rab3A interacting protein were down-regulated with 2.04-, 2.14-, 1.99-, 1.96-, 3.46-, 2.24-fold decreases.

Genes encoding vacuolar protein sorting proteins were down-regulated. Specifically the vacuolar protein sorting protein 18 that is required for membrane docking/fusion reactions of late endosomes/lysosomes had a 5.11-fold decrease. One gene encoding Golgi associated gamma-adaptin-related protein 1 that plays a role in protein sorting and trafficking between the trans-Golgi network (TGN) and endosomes had 1.33-fold decrease. Seven members of the TBC1 domain family of proteins were also down-regulated, with fold change ranges from -2.66 to -1.05, including proteins that act as GTPase-activators for Rab1, 2 and other Rab family proteins (Supplementary Table S22).

**Supplementary Tables**

Table S1 Raw Illumina Hi-Seq sequencing reads. 17 cDNA libraries were sequenced and produced a total about 346.2 million reads (*Chromera* infected and uninfected control) in 3 time points; 4, 12, and 48h post infection. NA= no data. The absence of data for the 12 h negative control 1 is due to RNA quality issues and the low number of reads for *Chromera*-infected 2 and 3 and negative control 1at 48h are consequences of low RNA yield.

| RNAseq library | 04 h | 12 h | 48 h | Total #  Illumina reads |
| --- | --- | --- | --- | --- |
| *Chromera*-infected 1 | 26,204,349 | 25,557,352 | 26,530,067 | 78,291,768 |
| *Chromera*-infected 2 | 25,721,449 | 26,639,362 | 2,336,789 | 54,697,600 |
| *Chromera*-infected 3 | 31,698,400 | 23,800,967 | 2,663,580 | 58,162,947 |
| Negative control 1 | 22,430,563 | NA | 2,355,166 | 24,785,729 |
| Negative control 2 | 19,542,091 | 24,035,359 | 15,729,866 | 59,307,316 |
| Negative control 3 | 20,912,751 | 25,197,830 | 24,849,108 | 70,959,689 |
| Total # Illumina reads | 146,509,603 | 125,230,870 | 74,464,576 | 346,205,049 |

Table S2 Percent of Illumina reads successfully mapped onto the *Acropora digitifera* transcriptome. NA= not applicable.

| Illumina RNA-Seq libraries | 04 h | 12 h | 48 h |
| --- | --- | --- | --- |
| *Chromera*-infected 1 | 33.18% | 36.24% | 34.71% |
| *Chromera*- infected 2 | 32.98% | 32.40% | 30.08% |
| *Chromera*- infected 3 | 34.83% | 30.08% | 32.40% |
| Negative control 1 | 34.83% | NA | 35.48% |
| Negative control 2 | 35.53% | 39.09% | 34.50% |
| Negative control 3 | 36.02% | 35.87% | 38.93% |

Table S3 Down-regulated *A. digitifera* differentially expressed transcripts in *Chromera* infected-larvae at 4 h time point with adjusted *P* ≤ 0.05. Columns correspond to coral cluster name, best BLASTX result, E-value and the log_2_ fold-change values

| Transcript ID | Best BLASTX hit | E-Value | logFC |
| --- | --- | --- | --- |
| adi_EST_assem_337 | Hmcn1_hemicentin-1 *(Homo sapiens*; Q96RW7) | 3.92E-72 | -4.57 |
| adi_EST_assem_5098 | Mfrp_membrane frizzled-related protein (*Acropora digitifera*; aug_v2a.12459) | 1.40E-13 | -3.98 |
| adi_EST_assem_3535 | Cytochrome P450, Family 17, Subfamily A (*Gallus gallu*s; P12394) | 3.32E-130 | -3.26 |
| adi_EST_assem_6300 | Iod3_type iii iodothyronine deiodinase (*Gallus gallus*; O42412) | 3.12E-44 | -3.23 |
| adi_EST_assem_1403 | Gp2_pancreatic secretory granule membrane major glycoprotein gp2 *(Homo sapiens*; P55259) | 5.49E-25 | -2.72 |
| adi_EST_assem_634 | Hmcn1_humanhemicentin-1 *(Homo sapiens*; Q96RW7) | 5.12E-67 | -2.7 |
| adi_EST_assem_2593 | Bhmt1_danrebetaine--homocysteine s-methyltransferase 1 (*Danio rerio*; Q32LQ4) | 1.72E-153 | -2.5 |
| adi_EST_assem_707 | Apcd1_pelsiprotein apcdd1 *(Pelodiscus sinensis*; Q5R2J4) | 3.70E-73 | -2.18 |
| adi_EST_assem_5604 | CUB domain-containing protein (*Acropora digitifera*; aug_v2a.10941) | 6.00E-55 | -2.05 |
| adi_EST_assem_12932 | Ap2a_rattranscription factor ap-2-alpha (*Rattus norvegicus*; P58197) | 5.25E-78 | -1.86 |
| adi_EST_assem_6642 | Rho1_ashgogtp-binding protein rho1 (*Ashbya gossypii*; Q9HF54) | 1.87E-38 | -1.84 |
| adi_EST_assem_8360 | Hmcn1_humanhemicentin-1 (*Homo sapiens*; Q96RW7) | 6.97E-72 | -1.68 |
| adi_EST_assem_3607 | Mot12_xentrmonocarboxylate transporter 12 *(Xenopus tropicalis*; Q6P2X9) | 1.82E-13 | -1.65 |
| adi_EST_assem_995 | Asomp_acrmiacidic skeletal organic matrix protein (*Acropora millepora*; B3EWY7) | 2.64E-177 | -1.63 |
| adi_EST_assem_23303 | Mdga1_mousemam domain-containing glycosylphosphatidylinositol anchor protein 1 *(Mus musculus*; Q0PMG2) | 1.35E-18 | -1.54 |
| adi_EST_assem_149 | Fp_acrmifibronectin type iii domain-containing protein *(Acropora millepora*; B8VIW9) | 0 | -1.53 |
| adi_EST_assem_3889 | Cd151_humancd151 antigen *(Homo sapiens*; P48509) | 2.02E-35 | -1.42 |
| adi_EST_assem_796 | Cah2_humancarbonic anhydrase 2 (*Homo sapiens*; P00918) | 4.90E-72 | -1.34 |
| adi_EST_assem_1686 | Hem1_rat5-aminolevulinate mitochondrial (*Rattus norvegicus*; P13195) | 4.84E-172 | -1.28 |
| adi_EST_assem_11420 | Yrbe_bacsuuncharacterized oxidoreductase *(Bacillus subtilis*; O05389) | 4.11E-54 | -1.19 |

Table S4 Down-regulation of *A. digitifera* DEGs common to *Chromera* and *Symbiodinium* infections at the 4 h time point (adjusted *P* ≤ 0.05).

| Transcript ID | Best BLASTX hit | logFC *Chromera* infection vs control | logFC *Symbiodinium* infection vs control |
| --- | --- | --- | --- |
| adi_EST_assem_1403 | GP2_pancreatic secretory granule membrane major glycoprotein gp2  (*Homo sapines*; P55259) | -2.72 | -2.1 |
| adi_EST_assem_2593 | BHMT1_betaine--homocysteine s-methyltransferase 1  (*Danio rerio*; Q32LQ4) | -2.5 | -1.7 |
| adi_EST_assem_707 | APCD1_protein apcdd1 (*Pelodiscus sinensis*; Q5R2J4) | -2.18 | -1.3 |
| adi_EST_assem_3607 | MO12_monocarboxylate transporter 12  (*Xenopus tropicalis*; Q6P2X9) | -1.65 | -1.3 |

Table S5 Significant KEGG pathway enrichment amongst the set of down- regulated genes in *Chromera*-infected larvae at 48 h post infection with Benjamini-corrected *P* ≤ 0.05.

| Significant KEGG pathway | KEGG pathway ID | No. of genes | Fold Enrichment |
| --- | --- | --- | --- |
| *Regulation of actin cytoskeleton* | hsa04810 | 19 | 2.93 |
| *Focal adhesion* | hsa04510 | 18 | 2.87 |
| *ECM-receptor interaction* | hsa04512 | 12 | 3.19 |

Table S6 Genes involved in the KEGG pathway *regulation of actin cytoskeleton*

| Cluster ID | Swiss-Prot ID | Best BLASTX Hit | Species | E-value | logFC |
| --- | --- | --- | --- | --- | --- |
| adi_EST_assem_13215 | Q9Y2X7 | G protein-coupled receptor kinase interacting arfgap 1 | *Homo sapiens* | 0 | -2.26 |
| adi_EST_assem_3391 | P46940 | IQ motif containing gtpase activating protein 1 | *Homo sapiens* | 0 | -2.67 |
| adi_EST_assem_5250 | O75116 | Rho-associated, coiled-coil containing protein kinase 2 | *Homo sapiens* | 0 | -2.83 |
| adi_EST_assem_8750 | Q13009 | T-cell lymphoma invasion and metastasis 1 | *Homo sapiens* | 9.40E-148 | -2.32 |
| adi_EST_assem_8735 | P42768 | Wiskott-Aldrich syndrome (eczema-thrombocytopenia) | *Homo sapiens* | 1.38E-56 | -1.4 |
| adi_EST_assem_4349 | P25054 | Adenomatous polyposis coli | *Homo sapiens* | 3.40E-114 | -1.64 |
| adi_EST_assem_12712 | Q14185 | Dedicator of cytokinesis 1 | *Homo sapiens* | 0.00E+00 | -2.17 |
| adi_EST_assem_5341 | O60879 | Diaphanous homolog 2 (Drosophila) | *Homo sapiens* | 2.06E-106 | -2.4 |
| adi_EST_assem_7497 | Q8N8S7 | Enabled homolog (Drosophila) | *Homo sapiens* | 1.37E-43 | -1.58 |
| adi_EST_assem_900 | P11362 | Fibroblast growth factor receptor 1 | *Homo sapiens* | 5.07E-95 | -1.79 |
| adi_EST_assem_898 | P22607 | Fibroblast growth factor receptor 3 | *Homo sapiens* | 1.65E-99 | -2.3 |
| adi_EST_assem_2436 | P15311 | Hypothetical protein LOC100129652; ezrin | *Homo sapiens* | 4.03E-156 | -2.05 |
| adi_EST_assem_5356 | P53708 | Integrin, alpha 8 | *Homo sapiens* | 7.89E-82 | -2 |
| adi_EST_assem_128 | Q13797 | Integrin, alpha 9 | *Homo sapiens* | 1.83E-98 | -1.5 |
| adi_EST_assem_2138 | P05556 | Integrin, beta 1 (fibronectin receptor, beta polypeptide, antigen CD29 includes MDF2, MSK12) | *Homo sapiens* | 1.91E-158 | -1.4 |
| adi_EST_assem_3616 | P27986 | Phosphoinositide-3-kinase, regulatory subunit 1 (alpha) | *Homo sapiens* | 5.02E-87 | -1.21 |
| adi_EST_assem_659 | O14974 | Protein phosphatase 1, regulatory (inhibitor) subunit 12A | *Homo sapiens* | 2.00E-80 | -1.43 |
| adi_EST_assem_5223 | Q07889 | Son of sevenless homolog 1 (Drosophila) | *Homo sapiens* | 0 | -2.21 |
| adi_EST_assem_10072 | Q9UKW4 | Vav 3 guanine nucleotide exchange factor | *Homo sapiens* | 5.43E-32 | -2.89 |

Table S7 Genes involved in the KEGG pathway *ECM receptor interaction*

| Cluster ID | Swiss-Prot ID | Best BLASTX Hit | Species | E-value | logFC |
| --- | --- | --- | --- | --- | --- |
| adi_EST_assem_13417 | O00468 | Agrin | *Homo sapiens* | 8.92E-45 | -1.09 |
| adi_EST_assem_5774 | A6NMZ7 | Collagen type VI alpha 6 | *Homo sapiens* | 2.87E-67 | -2.88 |
| adi_EST_assem_2378 | P12107 | Collagen, type XI, alpha 1 | *Homo sapiens* | 1.30E-48 | -2.34 |
| adi_EST_assem_4678 | Q14118 | Dystroglycan 1 (dystrophin-associated glycoprotein 1) | *Homo sapiens* | 2.89E-27 | -2.37 |
| adi_EST_assem_5356 | P53708 | Integrin, alpha 8 | *Homo sapiens* | 7.89E-82 | -2 |
| adi_EST_assem_128 | Q13797 | Integrin, alpha 9 | *Homo sapiens* | 1.83E-98 | -1.5 |
| adi_EST_assem_2138 | P05556 | Integrin, beta 1 (fibronectin receptor, beta polypeptide, antigen CD29 includes MDF2, MSK12) | *Homo sapiens* | 1.91E-158 | -1.43 |
| adi_EST_assem_53 | P25391 | Laminin, alpha 1 | *Homo sapiens* | 2.73E-45 | -3.31 |
| adi_EST_assem_574 | P07942 | Laminin, beta 1 | *Homo sapiens* | 0.00E+00 | -1.89 |
| adi_EST_assem_576 | P55268 | Laminin, beta 2 (laminin S) | *Homo sapiens* | 8.13E-78 | -2.53 |

Table S8 Genes involved in the KEGG pathway *focal adhesion*

| Cluster ID | Swiss-Prot ID | | Best BLASTX Hit | | | Species | | E-value | | logFC | |
| --- | --- | --- | --- | --- | --- | --- | --- | --- | --- | --- | --- |
| adi_EST_assem_8781 | | Q13905 | | Rap guanine nucleotide exchange factor (GEF) 1 | *Homo sapiens* | | 1.50E-16 | | -1.8 | |  |
| adi_EST_assem_5250 | | O75116 | | Rho-associated, coiled-coil containing protein kinase 2 | *Homo sapiens* | | 0 | | -2.82 | |  |
| adi_EST_assem_5774 | | A6NMZ7 | | Collagen type VI alpha 6 | *Homo sapiens* | | 2.87E-67 | | -2.88 | |  |
| adi_EST_assem_2378 | | P12107 | | Collagen, type XI, alpha 1 | *Homo sapiens* | | 1.30E-48 | | -2.34 | |  |
| adi_EST_assem_12712 | | Q14185 | | Dedicator of cytokinesis 1 | *Homo sapiens* | | 0.00E+00 | | -2.17 | |  |
| adi_EST_assem_5356 | | P53708 | | Integrin, alpha 8 | *Homo sapiens* | | 7.89E-82 | | -2 | |  |
| adi_EST_assem_128 | | Q13797 | | Integrin, alpha 9 | *Homo sapiens* | | 1.83E-98 | | -1.5 | |  |
| adi_EST_assem_2138 | | P05556 | | Integrin, beta 1 (fibronectin receptor, beta polypeptide, antigen CD29 includes MDF2, MSK12) | *Homo sapiens* | | 1.91E-158 | | -1.43 | |  |
| adi_EST_assem_53 | | P25391 | | Laminin, alpha 1 | *Homo sapiens* | | 2.73E-45 | | -3.31 | |  |
| adi_EST_assem_574 | | P07942 | | Laminin, beta 1 | *Homo sapiens* | | 0.00E+00 | | -1.89 | |  |
| adi_EST_assem_576 | | P55268 | | Laminin, beta 2 (laminin S) | *Homo sapiens* | | 8.13E-78 | | -2.53 | |  |
| adi_EST_assem_3616 | | P27986 | | Phosphoinositide-3-kinase, regulatory subunit 1 (alpha) | *Homo sapiens* | | 5.02E-87 | | -1.21 | |  |
| adi_EST_assem_659 | | O14974 | | Protein phosphatase 1, regulatory (inhibitor) subunit 12A | *Homo sapiens* | | 2.00E-80 | | -1.43 | |  |
| adi_EST_assem_5223 | | Q07889 | | Son of sevenless homolog 1 (Drosophila) | *Homo sapiens* | | 0.00E+00 | | -2.21 | |  |
| adi_EST_assem_175 | | Q9Y490 | | Talin 1 | *Homo sapiens* | | 2.34E-161 | | -1.79 | |  |
| adi_EST_assem_10072 | | Q9UKW4 | | Vav 3 guanine nucleotide exchange factor | *Homo sapiens* | | 5.43E-32 | | -2.89 | |  |

Table S9 Gene Ontology terms in the Biological Processes (BP) category that are significantly enriched amongst the set of down-regulated genes in *Chromera* infection compared to control at 48h with corrected *P* ≤ 0.05

| Annotation term | Gene Ontology (GO) term | No. of genes | Fold Enrichment |
| --- | --- | --- | --- |
| Regulation of small GTPase mediated signal transduction | GO:0051056 | 72 | 2.06 |
| Regulation of transcription and RNA metabolic process | GO:0045449, GO:0006350, GO:0006355,GO:0051252 | **301** | 1.34 |
| Regulation of Rho protein signal transduction | GO:0035023 | 33 | 2.65 |
| Regulation of Ras protein signal transduction and Ras GTPase activity | GO:0046578, GO:0032318 | 57 | 2.03 |
| Phosphate, phosphorus metabolic processes, and phosphorylation | GO:0006796, GO:0006793, GO:0006468, GO:0016310 | 187 | 1.41 |
| Regulation of GTPase activity | GO:0043087 | 37 | 2.17 |
| Microtubule-based movement | GO:0007018 | 48 | 1.92 |
| Chromatin modification | GO:0016568 | 67 | 1.71 |
| Biological, cell adhesion | GO:0022610, GO:0007155 | 104 | 1.51 |
| Transmembrane receptor protein tyrosine kinase signaling pathway | GO:0007169 | 44 | 1.95 |
| Cell projection organization | GO:0030030 | 80 | 1.58 |
| Microtubule-based process | GO:0007017 | 72 | 1.51 |
| Intracellular signaling cascade | GO:0007242 | 163 | 1.28 |
| Enzyme linked receptor protein signaling pathway | GO:0007167 | 53 | 1.61 |
| Neuron development, differentiation | GO:0030182, GO:0048666, GO:0031175 | 79 | 1.45 |
| Regulation of hydrolase activity | GO:0051336 | 47 | 1.62 |
| Detection of stimulus involved in sensory perception | GO:0050906 | 12 | 2.95 |

Table S10 Gene Ontology terms in the Cellular Component (CC) category that are significantly enriched amongst the set of down-regulated genes in *Chromera* infection compared to control at 48h with corrected *P* ≤ 0.05

| Annotation Term | Gene Ontology (GO) ID | No. of genes | Fold  Enrichment |
| --- | --- | --- | --- |
| Cytoskeleton, cytoskeletal part, microtubule, microtubule cytoskeleton, microtubule organizing center | GO:0005856, GO:0044430, GO:0005874, GO:0015630, GO:0005815, GO:0005874, GO:0005875 | 251 | 1.64 |
| Extrinsic to membrane | GO:0019898 | 116 | 1.70 |
| Cell projection | GO:0042995 | 127 | 1.61 |
| Intracellular non-membrane-bounded organelle | GO:0043232, GO:0043228 | 394 | 1.25 |
| Centrosome | GO:0005813 | 58 | 1.79 |
| Plasma membrane | GO:0005886, GO:0044459, GO:0005624 | 371 | 1.19 |
| Cell fraction | GO:0000267 | 127 | 1.40 |
| Cell junction | GO:0030054 | 74 | 1.54 |
| Cell projection part | GO:0044463 | 54 | 1.68 |
| Neuron projection | GO:0043005 | 61 | 1.60 |
| Adherens junction | GO:0005912 | 27 | 2.04 |
| Insoluble fraction | GO:0005626 | 105 | 1.38 |
| Anchoring junction | GO:0070161 | 27 | 2.01 |
| Cell leading edge | GO:0031252 | 29 | 1.93 |
| Nuclear envelope | GO:0005635 | 45 | 1.66 |
| Nuclear lumen | GO:0031981 | 199 | 1.23 |
| Perinuclear region of cytoplasm | GO:0048471 | 44 | 1.66 |
| Extracellular matrix part | GO:0044420 | 33 | 1.81 |
| Cell cortex | GO:0005938 | 29 | 1.87 |
| Ruffle | GO:0001726 | 15 | 2.53 |
| Cilium | GO:0005929 | 37 | 1.71 |
| Axon | GO:0030424 | 30 | 1.83 |
| Lamellipodium | GO:0030027 | 18 | 2.26 |
| Actin cytoskeleton | GO:0015629 | 42 | 1.63 |
| Axoneme | GO:0005930 | 20 | 2.14 |
| Vesicle | GO:0031982 | 101 | 1.34 |
| DNA-directed RNA polymerase II, holoenzyme | GO:0016591 | 25 | 1.92 |
| Cell-cell junction | GO:0005911 | 23 | 1.98 |
| Nucleoplasm part | GO:0044451 | 91 | 1.35 |
| Dendrite | GO:0030425 | 31 | 1.72 |
| Nuclear pore | GO:0005643 | 24 | 1.85 |
| Cytoplasmic vesicle | GO:0031410 | 95 | 1.30 |
| Pore complex | GO:0046930 | 25 | 1.77 |
| Basolateral plasma membrane | GO:0016323 | 29 | 1.67 |

Table S11 Gene Ontology terms in the Molecular Function (MF) category that are significantly enriched amongst the set of down-regulated genes in *Chromera* infection compared to control at 48h with corrected *P* ≤ 0.05

| Annotation Term | Gene Ontology (GO) IDs | No. of genes | Fold Enrichment |
| --- | --- | --- | --- |
| GTPase regulator activity | GO:0030695, GO:0060589, GO:0005083, GO:0005096 | 122 | 2.33 |
| Guanyl-nucleotide exchange factor activity | GO:0005085 | 54 | 2.51 |
| Protein kinase activity | GO:0004672 | 150 | 1.58 |
| Enzyme activator activity | GO:0008047 | 70 | 2.01 |
| Rho guanyl-nucleotide exchange factor activity | GO:0005089 | 32 | 2.81 |
| Calcium ion binding | GO:0005509 | 198 | 1.45 |
| Ras guanyl-nucleotide exchange factor activity | GO:0005088 | 34 | 2.7 |
| Nucleotide binding, purine nucleotide binding, ribonucleotide binding, adenyl nucleotide binding | GO:0000166, GO:0017076, GO:0032555, GO:0001883, GO:0030554, GO:0001882, GO:0005524, GO:0032559 | 498 | 1.21 |
| Motor activity | GO:0003774, GO:0003777 | 59 | 1.95 |
| Gtpase binding, small gtpase binding | GO:0051020, GO:0031267 | 29 | 2.5 |
| Enzyme binding | GO:0019899 | 78 | 1.65 |
| Ras gtpase activator activity, Ras gtpase binding, | GO:0005099, GO:0017016 | 29 | 2.69 |
| DNA binding | GO:0003677 | 261 | 1.25 |
| Cytoskeletal protein binding | GO:0008092 | 87 | 1.51 |
| Transcription regulator activity | GO:0030528 | 165 | 1.33 |
| Atpase activity | GO:0016887 | 104 | 1.44 |
| Ion binding, metal ion binding | GO:0043167, GO:0043169, GO:0046872 | 673 | 1.11 |
| Protein domain specific binding | GO:0019904 | 55 | 1.65 |
| Calmodulin binding | GO:0005516 | 40 | 1.78 |
| Protein serine/threonine kinase activity | GO:0004674, GO:0004713, GO:0004714 | 100 | 1.4 |
| Diacylglycerol binding | GO:0019992 | 22 | 2.22 |
| Actin binding, actin filament binding | GO:0003779, GO:0051015 | 62 | 1.54 |
| Transcription factor binding | GO:0008134 | 60 | 1.46 |
| Rab gtpase activator activity | GO:0005097, GO:0017137 | 18 | 2.12 |
| Zinc ion binding | GO:0008270 | 322 | 1.14 |
| SH3 domain binding | GO:0017124 | 21 | 1.93 |

Table S12 Annotations of the down-regulated *A. digitifera* transcripts involved in GTPase regulator activity in *Chromera*-infected larvae at 48 h post infection with corrected *P* ≤ 0.05

| ID | Protein Name | Species |
| --- | --- | --- |
| Q12802 | A kinase (PRKA) anchor protein 13 | *Homo sapiens* |
| Q9FN03 | At5g63860 | *Arabidopsis thaliana* |
| P52594 | Arfgap with FG repeats 1 | *Homo sapiens* |
| Q4LDD4 | Arfgap with rhogap domain, ankyrin repeat and PH domain 1 | *Mus musculus* |
| O97902 | Arfgap with SH3 domain, ankyrin repeat and PH domain 1 | *Bos taurus* |
| Q96P50 | Arfgap with coiled-coil, ankyrin repeat and PH domains 3 | *Homo sapiens* |
| O54874 | CDC42 binding protein kinase alpha | *Rattus norvegicus* |
| Q8TDJ6 | Dmx-like 2 | *Homo sapiens* |
| Q96EY1 | Dnaj (Hsp40) homolog, subfamily A, member 3 | *Homo sapiens* |
| Q8IUD2 | ELKS/RAB6-interacting/CAST family member 1 | *Homo sapiens* |
| Q91VS8 | FERM, rhogef and pleckstrin domain protein 2 | *Mus musculus* |
| P52734 | FYVE, rhogef and PH domain containing 1 | *Mus musculus* |
| Q96M96 | FYVE, rhogef and PH domain containing 4 | *Homo sapiens* |
| Q6ZV73 | FYVE, rhogef and PH domain containing 6 | *Homo sapiens* |
| Q9Y2X7 | G protein-coupled receptor kinase interacting arfgap 1 | *Homo sapiens* |
| P81274 | G-protein signaling modulator 2 (AGS3-like, C. Elegans) | *Homo sapiens* |
| Q5VVW2 | Gtpase activating Rap/rangap domain-like 3 | *Homo sapiens* |
| P33277 | Gtpase-activating protein | *Schizosaccharomyces pombe* |
| Q8R0S2 | IQ motif and Sec7 domain 1 | *Mus musculus* |
| P46940 | IQ motif containing gtpase activating protein 1 | *Homo sapiens* |
| Q86X10 | Kiaa1219 | *Homo sapiens* |
| Q5TH69 | Kiaa1244 | *Homo sapiens* |
| Q80U28 | MAP-kinase activating death domain | *Mus musculus* |
| O15068 | MCF.2 cell line derived transforming sequence-like | *Homo sapiens* |
| Q6GPD0 | MGC80493 protein | *Xenopus laevis* |
| Q28C33 | Novel protein containing TBC domain domain | *Xenopus tropicalis* |
| Q5ZJ17 | RAB gtpase activating protein 1-like | *Gallus gallus* |
| O75154 | RAB11 family interacting protein 3 (class II) | *Homo sapiens* |
| Q15042 | RAB3 gtpase activating protein subunit 1 (catalytic) | *Homo sapiens* |
| Q8BMG7 | RAB3 gtpase activating protein subunit 2 | *Mus musculus* |
| Q9H2M9 | RAB3 gtpase activating protein subunit 2 (non-catalytic) | *Homo sapiens* |
| Q62739 | RAB3A interacting protein (rabin3) | *Rattus norvegicus* |
| P47736 | RAP1 gtpase activating protein | *Homo sapiens* |
| Q8IV61 | RAS guanyl releasing protein 3 (calcium and DAG-regulated) | *Homo sapiens* |
| P28818 | RAS protein-specific guanine nucleotide-releasing factor 1 | *Rattus norvegicus* |
| A1IGU4 | RIKEN cdna 4933429F08 gene | *Mus musculus* |
| Q8BQZ4 | RIKEN cdna B230339M05 gene | *Mus musculus* |
| Q6QI06 | RPTOR independent companion of MTOR, complex 2 | *Mus musculus* |
| Q13905 | Rap guanine nucleotide exchange factor (GEF) 1 | *Homo sapiens* |
| Q8TEU7 | Rap guanine nucleotide exchange factor (GEF) 6 | *Homo sapiens* |
| Q8JZL7 | Rasgef domain family, member 1B; hypothetical protein LOC100044232 | *Mus musculus* |
| Q8IWW6 | Rho gtpase activating protein 12 | *Homo sapiens* |
| Q8K0Q5 | Rho gtpase activating protein 18 | *Mus musculus* |
| Q6REY9 | Rho gtpase activating protein 20 | *Rattus norvegicus* |
| Q5T5U3 | Rho gtpase activating protein 21 | *Homo sapiens* |
| Q7Z5H3 | Rho gtpase activating protein 22 | *Homo sapiens* |
| Q8N264 | Rho gtpase activating protein 24 | *Homo sapiens* |
| Q9UNA1 | Rho gtpase activating protein 26 | *Homo sapiens* |
| P97393 | Rho gtpase activating protein 5 | *Mus musculus* |
| O43182 | Rho gtpase activating protein 6 | *Homo sapiens* |
| Q9V9S7 | Rho gtpase-activating protein 100F | *Drosophila melanogaster* |
| Q8C033 | Rho guanine nucleotide exchange factor (GEF) 10 | *Mus musculus* |
| O15085 | Rho guanine nucleotide exchange factor (GEF) 11 | *Homo sapiens* |
| Q96PE2 | Rho guanine nucleotide exchange factor (GEF) 17 | *Homo sapiens* |
| Q8N5H7 | SH2 domain containing 3C | *Homo sapiens* |
| Q13009 | T-cell lymphoma invasion and metastasis 1 | *Homo sapiens* |
| Q5F361 | TBC domain-containing protein kinase-like | *Gallus gallus* |
| Q86TI0 | TBC1 (tre-2/USP6, BUB2, cdc16) domain family, member 1 | *Homo sapiens* |
| Q8C9V1 | TBC1 domain family, member 10c | *Mus musculus* |
| A6H7I8 | TBC1 domain family, member 14 | *Bos taurus* |
| Q9D9I4 | TBC1 domain family, member 20 | *Mus musculus* |
| Q9NU19 | TBC1 domain family, member 22B | *Homo sapiens* |
| Q9UPU7 | TBC1 domain family, member 2B | *Homo sapiens* |
| Q92609 | TBC1 domain family, member 5 | *Homo sapiens* |
| P83510 | TRAF2 and NCK interacting kinase | *Mus musculus* |
| Q92738 | USP6 N-terminal like | *Homo sapiens* |
| Q6NXY1 | WD repeat domain 67 | *Mus musculus* |
| P42768 | Wiskott-Aldrich syndrome (eczema-thrombocytopenia) | *Homo sapiens* |
| A6QNS3 | Active BCR-related gene | *Bos taurus* |
| Q96Q42 | Amyotrophic lateral sclerosis 2 (juvenile) | *Homo sapiens* |
| P0C5Y8 | Amyotrophic lateral sclerosis 2 (juvenile) homolog (human) | *Rattus norvegicus* |
| Q920R0 | Amyotrophic lateral sclerosis 2 (juvenile) homolog (human) | *Mus musculus* |
| Q3UMR0 | Ankyrin repeat domain 27 (VPS9 domain) | *Mus musculus* |
| P11274 | Breakpoint cluster region | *Homo sapiens* |
| Q2PPJ7 | Chromosome 20 open reading frame 74 | *Homo sapiens* |
| O14578 | Citron (rho-interacting, serine/threonine kinase 21) | *Homo sapiens* |
| Q14185 | Dedicator of cytokinesis 1 | *Homo sapiens* |
| Q8BUR4 | Dedicator of cytokinesis 1 | *Mus musculus* |
| Q8R1A4 | Dedicator of cytokinesis 7 | *Mus musculus* |
| Q8BIK4 | Dedicator of cytokinesis 9 | *Mus musculus* |
| Q9BZ29 | Dedicator of cytokinesis 9 | *Homo sapiens* |
| Q6XZF7 | Dynamin binding protein | *Homo sapiens* |
| Q8CHW4 | Eukaryotic translation initiation factor 2B, subunit 5 epsilon | *Mus musculus* |
| Q8TBA6 | Golgi autoantigen, golgin subfamily a, 5 | *Homo sapiens* |
| Q15751 | Hect (homologous to the E6-AP (UBE3A) carboxyl terminus) domain and RCC1 (CHC1)-like domain (RLD) 1 | *Homo sapiens* |
| O95714 | Hect domain and RLD 2 | *Homo sapiens* |
| Q92619 | Histocompatibility (minor) HA-1 | *Homo sapiens* |
| Q9PT60 | Hypothetical protein MGC81374 | *Xenopus laevis* |
| O00410 | Importin 5 | *Homo sapiens* |
| O95373 | Importin 7 | *Homo sapiens* |
| Q9NZM3 | Intersectin 2 | *Homo sapiens* |
| Q9Z0R6 | Intersectin 2 | *Mus musculus* |
| Q76NI1 | Kinase non-catalytic C-lobe domain (KIND) containing 1 | *Homo sapiens* |
| Q5S007 | Leucine-rich repeat kinase 2 | *Homo sapiens* |
| Q8BPM2 | Mitogen-activated protein kinase kinase kinase kinase 5 | *Mus musculus* |
| B2RTY4 | Myosin IXA | *Homo sapiens* |
| Q13459 | Myosin IXB | *Homo sapiens* |
| P70569 | Myosin Vb | *Rattus norvegicus* |
| P97526 | Neurofibromin 1 | *Rattus norvegicus* |
| Q5BKC9 | Neuronal guanine nucleotide exchange factor | *Rattus norvegicus* |
| Q9ULL1 | Pleckstrin homology domain containing, family G (with rhogef domain) member 1 | *Homo sapiens* |
| Q58EX7 | Pleckstrin homology domain containing, family G (with rhogef domain) member 4 | *Homo sapiens* |
| O94827 | Pleckstrin homology domain containing, family G (with rhogef domain) member 5 | *Homo sapiens* |
| Q60695 | Ral guanine nucleotide dissociation stimulator,-like 1 | *Mus musculus* |
| Q9JIS1 | Regulating synaptic membrane exocytosis 2 | *Rattus norvegicus* |
| Q8CGE9 | Regulator of G-protein signaling 12 | *Mus musculus* |
| Q92834 | Retinitis pigmentosa gtpase regulator | *Homo sapiens* |
| Q5RHR6 | Si:dkey-233p4.1 | *Danio rerio* |
| P69735 | Similar to RAB3 gtpase-activating protein | *Rattus norvegicus* |
| O43147 | Small G protein signaling modulator 2 | *Homo sapiens* |
| Q96HU1 | Small G protein signaling modulator 3 | *Homo sapiens* |
| Q07889 | Son of sevenless homolog 1 (Drosophila) | *Homo sapiens* |
| Q9R0X5 | Sushi-repeat-containing protein; retinitis pigmentosa gtpase regulator | *Mus musculus* |
| Q96C24 | Synaptotagmin-like 4 | *Homo sapiens* |
| Q8WZ42 | Titin | *Homo sapiens* |
| O75962 | Triple functional domain (PTPRF interacting) | *Homo sapiens* |
| Q0KL02 | Triple functional domain (PTPRF interacting) | *Mus musculus* |
| P49815 | Tuberous sclerosis 2 | *Homo sapiens* |
| Q07912 | Tyrosine kinase, non-receptor, 2 | *Homo sapiens* |
| Q8R5L3 | Vacuolar protein sorting 39 (yeast) | *Mus musculus* |
| Q60992 | Vav 2 oncogene | *Mus musculus* |
| Q9UKW4 | Vav 3 guanine nucleotide exchange factor | *Homo sapiens* |

Table S13 Gene Ontology terms in the Biological Processes (BP) category that are significantly enriched amongst the set of up-regulated genes in *Chromera* infection compared to control at 48h with corrected *P* ≤ 0.05

| Annotation term | Gene Ontology  (GO) ID | No. of genes | Fold Enrichment |
| --- | --- | --- | --- |
| Translation | GO:0006412 | 63 | 2.19 |
| Electron transport chain | GO:0022900 | 28 | 2.97 |
| Protein targeting to membrane | GO:0006612 | 11 | 6.95 |
| Protein transport, localization | GO:0008104, GO:0045184,  GO:0015031 | 80 | 1.57 |
| Protein import into mitochondrial inner membrane | GO:0045039,  GO:0007007 | 6 | 10.74 |
| Oxidative phosphorylation | GO:0006119 | 14 | 3.41 |
| Intracellular transport | GO:0046907 | 54 | 1.68 |
| Mitochondrial membrane organization | GO:0007006 | 9 | 5.08 |
| Mitochondrion organization | GO:0007005 | 17 | 2.8 |
| Intracellular protein transport | GO:0006886 | 35 | 1.89 |
| Protein targeting | GO:0006605 | 22 | 2.3 |

Table S14 Gene Ontology terms in the Cellular Component (CC) category that are significantly enriched amongst the set of up-regulated genes in *Chromera* infection compared to control at 48h with corrected *P* ≤ 0.05

| Annotation Term | Gene Ontology (GO) IDs | No. of genes | Fold Enrichment |
| --- | --- | --- | --- |
| Mitochondrion | GO:0005739, GO:0044429 | 165 | 2.19 |
| Ribosome | GO:0005840 | 54 | 2.77 |
| Mitochondrial envelope/ membrane | GO:0005740, GO:0031966 | 64 | 2.44 |
| Ribonucleoprotein complex | GO:0030529 | 81 | 2 |
| Mitochondrial inner membrane | GO:0005743, GO:0044455 | 49 | 2.51 |
| Organelle membrane | GO:0031090, GO:0019866, GO:0031967 | 100 | 1.73 |
| Respiratory chain, Respiratory chain complex I,  NADH dehydrogenase complex | GO:0070469, GO:0045271,  GO:0030964 | 18 | 4.67 |
| Mitochondrial ribosome | GO:0005761 | 13 | 5.87 |
| Organellar ribosome | GO:0000313 | 13 | 5.87 |
| Envelope | GO:0031975 | 70 | 1.79 |
| Ribosomal subunit | GO:0033279 | 24 | 2.93 |
| Mitochondrial inter-membrane space | GO:0005758, GO:0042719 | 12 | 4.61 |
| Mitochondrial small ribosomal subunit | GO:0005763, GO:0000314, GO:0016272 | 6 | 7.78 |
| Srb-mediator complex | GO:0016592 | 9 | 4.45 |
| Organelle envelope lumen | GO:0031970 | 12 | 3.36 |
| Mitochondrial matrix and lumen | GO:0005759, GO:0031980 | 27 | 1.94 |
| Small ribosomal subunit | GO:0015935 | 12 | 3.03 |
| Proteasome core complex | GO:0005839 | 6 | 5.66 |
| Endoplasmic reticulum | GO:0005783 | 72 | 1.38 |
| Large ribosomal subunit | GO:0015934 | 12 | 2.7 |
| Mitochondrial respiratory chain | GO:0005746 | 6 | 5.19 |
| Mitochondrial large ribosomal subunit | GO:0005762, GO:0000315 | 6 | 5.19 |
| Organellar large ribosomal subunit |  |  |  |
| Mitochondrial respiratory chain complex I | GO:0005747 | 5 | 6.49 |
| DNA-directed RNA polymerase II, core complex | GO:0005665 | 5 | 6.49 |

Table S15 Gene Ontology terms in the Molecular Function (MF) category that are significantly enriched amongst the set of up-regulated genes in *Chromera* infection compared to control at 48h with corrected *P* ≤ 0.05

| Annotation term | Gene Ontology (GO) ID | No. of genes | Fold Enrichment |
| --- | --- | --- | --- |
| Structural constituent of ribosome | GO:0003735 | 50 | 3.32 |
| Structural molecule activity | GO:0005198 | 54 | 2 |
| Peptidyl-prolyl cis-trans isomerase activity | GO:0003755 | 10 | 4.5 |
| RNA polymerase II transcription mediator activity | GO:0016455 | 9 | 5 |
| Cis-trans isomerase activity | GO:0016859 | 10 | 4.34 |
| General RNA polymerase II transcription factor activity | GO:0016251 | 10 | 4.18 |
| Hydrogen ion transmembrane transporter activity | GO:0015078 | 13 | 3.17 |

Table S16 Annotations of the up-regulated *A. digitifera* transcripts involved in ribosome functions and translation in *Chromera*-infected larvae at 48 h post infection with corrected *P* ≤ 0.05

| Uni-Prot ID | Protein Name | Species |
| --- | --- | --- |
| Q6NTS3 | 28S ribosomal protein S24-B, mitochondrial | *Xenopus laevis* |
| A6U882 | 30S ribosomal protein S11 | *Sinorhizobium medicae* |
| Q9FNP8 | 40S ribosomal protein S19-3 | *Arabidopsis thaliana* |
| B3CRZ0 | 50S ribosomal protein L20 | *Orientia tsutsugamushi* |
| A0LFC4 | 50S ribosomal protein L35 | *Syntrophobacter fumaroxidans* |
| O14464 | 54S ribosomal protein YPL183W-A, mitochondrial | *Saccharomyces cerevisiae* |
| Q95WA0 | 60S ribosomal protein L26 | *Littorina littorea* |
| Q9NB33 | 60S ribosomal protein L44 | *Ochlerotatus triseriatus* |
| C4KZM8 | Eat1b_1615 | *Exiguobacterium sp. AT1b* |
| A7SGZ5 | Eukaryotic translation initiation factor 3 subunit K | *Nematostella vectensis* |
| A7SPW6 | NEMVEDRAFT_v1g215604 | *Nematostella vectensis* |
| P42678 | Protein translation factor SUI1 homolog | *Anopheles gambiae* |
| P49180 | Ribosomal Protein, Large subunit | *Caenorhabditis elegans* |
| P36241 | Ribosomal protein L19 | *Drosophila melanogaster* |
| Q5ZJ39 | Density-regulated protein | *Gallus gallus* |
| O70251 | Eukaryotic translation elongation factor 1 beta 2 | *Mus musculus* |
| P70541 | Eukaryotic translation initiation factor 2B, subunit 3 gamma | *Rattus norvegicus* |
| Q13542 | Eukaryotic translation initiation factor 4E binding protein 2 | *Homo sapiens* |
| O60573 | Eukaryotic translation initiation factor 4E family member 2 | *Homo sapiens* |
| P67985 | Heparin binding protein | *Sus scrofa* |
| Q8R035 | Immature colon carcinoma cluster 1 | *Mus musculus* |
| Q3TBW2 | Mitochondrial ribosomal protein L10 | *Mus musculus* |
| Q9D1P0 | Mitochondrial ribosomal protein L13 | *Mus musculus* |
| Q9D1I6 | Mitochondrial ribosomal protein L14 | *Mus musculus* |
| Q2TBI6 | Mitochondrial ribosomal protein L32 | *Bos taurus* |
| Q9CQP0 | Mitochondrial ribosomal protein L33 | *Mus musculus* |
| Q9DCU6 | Mitochondrial ribosomal protein L4 | *Mus musculus* |
| Q6DJI4 | Mitochondrial ribosomal protein L41 | *Xenopus laevis* |
| Q08DT6 | Mitochondrial ribosomal protein L47 | *Bos taurus* |
| Q9CQ40 | Mitochondrial ribosomal protein L49; similar to mitochondrial ribosomal protein L49 | *Mus musculus* |
| Q9VE04 | Mitochondrial ribosomal protein L55 | *Drosophila melanogaster* |
| Q9VFB2 | Mitochondrial ribosomal protein S10 | *Drosophila melanogaster* |
| O35680 | Mitochondrial ribosomal protein S12 | *Mus musculus* |
| Q9CR88 | Mitochondrial ribosomal protein S14; similar to mitochondrial ribosomal protein S14 | *Mus musculus* |
| Q9V6Y3 | Mitochondrial ribosomal protein S16 | *Drosophila melanogaster* |
| Q99N85 | Mitochondrial ribosomal protein S18A | *Mus musculus* |
| Q767K8 | Mitochondrial ribosomal protein S18B | *Sus scrofa* |
| Q8R2L5 | Mitochondrial ribosomal protein S18C | *Mus musculus* |
| P82920 | Mitochondrial ribosomal protein S21 | *Bos taurus* |
| Q9VZD5 | Mitochondrial ribosomal protein S6 | *Drosophila melanogaster* |
| Q9HBH1 | Peptide deformylase (mitochondrial); component of oligomeric golgi complex 8 | *Homo sapiens* |
| Q9Y3E5 | Peptidyl-trna hydrolase 2 | *Homo sapiens* |
| P51410 | Predicted gene 10117; similar to ribosomal protein L9; ribosomal protein L9 | *Mus musculus* |
| O09167 | Predicted gene 12618; predicted gene 8724; predicted gene 10155; predicted gene 3355; predicted gene 3713; predicted gene 3201; predicted gene 13641; similar to ribosomal protein L21 | *Mus musculus* |
| O35972 | Predicted gene 13671; mitochondrial ribosomal protein L23 | *Mus musculus* |
| Q7ZWJ4 | Ribosomal protein l18a | *Danio rerio* |
| P47830 | Ribosomal protein L22 | *Xenopus laevis* |
| Q8JGR4 | Ribosomal protein L24 | *Danio rerio* |
| P17078 | Ribosomal protein L35; similar to 60S ribosomal protein L35 | *Rattus norvegicus* |
| P62282 | Ribosomal protein S11 | *Rattus norvegicus* |
| P84175 | Ribosomal protein S12 | *Gallus gallus* |
| P62268 | Ribosomal protein S23; similar to ribosomal protein S23 | *Rattus norvegicus* |
| P62247 | Ribosomal protein S8 | *Danio rerio* |
| P47826 | Ribosomal protein, large, P0 | *Gallus gallus* |
| P23358 | Similar to 60S ribosomal protein L12; ribosomal protein L12 | *Rattus norvegicus* |
| P24049 | Similar to 60S ribosomal protein L17 (L23); similar to 60S ribosomal protein L17 (L23) (Amino acid starvation-induced protein) (ASI); ribosomal protein L17; hypothetical gene supported by X60212 | *Rattus norvegicus* |
| Q767K8 | Similar to mitochondrial ribosomal protein S18-2 | *Sus scrofa* |
| Q767K8 | Similar to mitochondrial ribosomal protein S18-2 | *Sus scrofa* |
| P62278 | Similar to ribosomal protein S13; ribosomal protein S13 | *Rattus norvegicus* |
| P23403 | Similar to ribosomal protein S20 | *Xenopus laevis* |

Table S17 Annotations of the up-regulated *A. digitifera* transcripts involved in mitochondrial functions in *Chromera*-infected larvae at 48 h post infection with corrected *P* ≤ 0.05

| ID | Protein Name | Species |
| --- | --- | --- |
| Q6NTS3 | 28S ribosomal protein S24-B, mitochondrial | *Xenopus laevis* |
| O14464 | 54S ribosomal protein YPL183W-A, mitochondrial | *Saccharomyces cerevisiae* |
| Q8VZF6 | AT5G45560 | *Arabidopsis thaliana* |
| O75964 | ATP synthase, H+ transporting, mitochondrial F0 complex, subunit G | *Homo sapiens* |
| P22027 | ATP synthase, H+ transporting, mitochondrial F0 complex, subunit s (factor B) | *Bos taurus* |
| Q00709 | B-cell CLL/lymphoma 2 | *Gallus gallus* |
| P0C7P0 | CDGSH iron sulfur domain 3 | *Homo sapiens* |
| A0JNC1 | CDP-diacylglycerol synthase (phosphatidate cytidylyltransferase) 2 | *Bos taurus* |
| P06197 | CDP-diacylglycerol--inositol 3-phosphatidyltransferase | *Saccharomyces cerevisiae* |
| Q3SZM6 | COX assembly mitochondrial protein homolog (S. cerevisiae) | *Bos taurus* |
| Q2NKS2 | COX16 cytochrome c oxidase assembly homolog (S. cerevisiae) | *Bos taurus* |
| Q6J3Q7 | COX17 homolog, cytochrome c oxidase assembly protein (yeast) | *Canis lupus* |
| Q8VC74 | COX18 cytochrome c oxidase assembly homolog (S. cerevisiae) | *Mus musculus* |
| Q5FVL2 | COX4 neighbor | *Rattus norvegicus* |
| B0XK69 | CpipJ_CPIJ019830 | *Culex quinquefasciatus* |
| Q6QLW4 | Cytochrome c | *Pectinaria gouldii* |
| Q3ZBN8 | DnaJ (Hsp40) homolog, subfamily C, member 19 | *Bos taurus* |
| P37193 | Ferredoxin | *Drosophila melanogaster* |
| Q5RDW1 | GTP binding protein 5 (putative) | *Pongo abelii* |
| Q9N121 | H protein | *Oryctolagus cuniculus* |
| Q9D7P6 | IscU iron-sulfur cluster scaffold homolog (E. coli); similar to nitrogen fixation cluster-like | *Mus musculus* |
| O15091 | KIAA0391 | *Homo sapiens* |
| Q8K215 | LYR motif containing 4 | *Mus musculus* |
| Q91V16 | LYR motif containing 5 | *Mus musculus* |
| Q6GR66 | MGC78819 protein | *Xenopus laevis* |
| Q6DFN1 | MGC79777 protein | *Xenopus (Silurana) tropicalis* |
| Q68EV6 | MGC84279 protein | *Xenopus laevis* |
| Q6DDX7 | MGC84796 protein | *Xenopus laevis* |
| Q66L32 | MGC85218 protein | *Xenopus laevis* |
| Q4P4Y2 | Mitochondrial genome maintenance protein MGM101 | *Ustilago maydis* |
| Q9Y1A3 | Mitochondrial import inner membrane translocase subunit Tim8 | *Drosophila melanogaster* |
| Q39056 | Molybdopterin biosynthesis protein CNX3 | *Arabidopsis thaliana* |
| Q2KIN6 | MpV17 mitochondrial inner membrane protein | *Bos taurus* |
| Q95KV7 | NADH dehydrogenase (ubiquinone) 1 alpha subcomplex, 13 | *Bos taurus* |
| O43678 | NADH dehydrogenase (ubiquinone) 1 alpha subcomplex, 2, 8kDa | *Homo sapiens* |
| Q6PBH5 | NADH dehydrogenase (ubiquinone) 1 alpha subcomplex, 4; hypothetical LOC799961 | *Danio rerio* |
| P23935 | NADH dehydrogenase (ubiquinone) 1 alpha subcomplex, 5, 13kDa | *Bos taurus* |
| Q05752 | NADH dehydrogenase (ubiquinone) 1 alpha subcomplex, 7, 14.5kDa | *Bos taurus* |
| Q0MQB1 | NADH dehydrogenase (ubiquinone) 1 alpha subcomplex, 8, 19kDa | *Pan troglodytes* |
| Q5BK63 | NADH dehydrogenase (ubiquinone) 1 alpha subcomplex, 9 | *Rattus norvegicus* |
| Q02365 | NADH dehydrogenase (ubiquinone) 1 beta subcomplex, 3, 12kDa | *Bos taurus* |
| Q0MQF0 | NADH dehydrogenase (ubiquinone) 1 beta subcomplex, 9, 22kDa | *Pan troglodytes* |
| P23709 | NADH dehydrogenase (ubiquinone) Fe-S protein 3, 30kDa (NADH-coenzyme Q reductase) | *Bos taurus* |
| Q5XIF3 | NADH dehydrogenase (ubiquinone) Fe-S protein 4 | *Rattus norvegicus* |
| P52504 | NADH dehydrogenase (ubiquinone) Fe-S protein 6 | *Rattus norvegicus* |
| Q9M9B4 | NADH dehydrogenase [ubiquinone] iron-sulfur protein 6 | *Arabidopsis thaliana* |
| Q86UD5 | Na+/H+ exchanger domain containing 2 | *Homo sapiens* |
| Q24439 | Oligomycin sensitivity-conferring protein | *Drosophila melanogaster* |
| Q9DAK2 | PARK2 co-regulated | *Mus musculus* |
| Q90673 | PRELI domain containing 1 | *Gallus gallus* |
| Q86BN8 | PTEN-like phosphatase | *Drosophila melanogaster* |
| Q8UW59 | Parkinson disease (autosomal recessive, early onset) 7 | *Gallus gallus* |
| Q9VAI1 | Probable complex I intermediate-associated protein 30, mitochondrial | *Drosophila melanogaster* |
| A7S1H9 | Protein ACN9 homolog, mitochondrial | *Nematostella vectensis* |
| Q66GV0 | Protein Mpv17 | *Xenopus laevis* |
| A8WGF7 | Protein spinster homolog 1 | *Xenopus (Silurana) tropicalis* |
| Q9CR10 | RIKEN cDNA 1810049H13 gene | *Mus musculus* |
| Q8C1Q6 | RIKEN cDNA 2010107H07 gene | *Mus musculus* |
| Q7TNS2 | RIKEN cDNA 2310028O11 gene | *Mus musculus* |
| Q9CWB7 | RIKEN cDNA C330018D20 gene | *Mus musculus* |
| Q5SUC9 | SCO cytochrome oxidase deficient homolog 1 (yeast) | *Mus musculus* |
| P60924 | SEL1 domain containing protein RGD735029 | *Rattus norvegicus* |
| Q95KK4 | Sjogren syndrome antigen B (autoantigen La) | *Oryctolagus cuniculus* |
| Q9P7Q5 | Uncharacterized protein C1834.10c | *Schizosaccharomyces pombe* |
| Q9CYF5 | Williams-Beuren syndrome chromosome region 16 homolog (human) | *Mus musculus* |
| Q5R833 | Acyl-coa thioesterase 13 | *Pongo abelii* |
| O95881 | Apoptosis-inducing factor, mitochondrion-associated, 1 | *Homo sapiens* |
| O95563 | Brain protein 44 | *Homo sapiens* |
| P63031 | Brain protein 44-like; similar to brain protein 44-like | *Rattus norvegicus* |
| Q9Y259 | Choline kinase beta; carnitine palmitoyltransferase 1B (muscle) | *Homo sapiens* |
| Q9NZJ6 | Coenzyme Q3 homolog, methyltransferase (S. Cerevisiae) | *Homo sapiens* |
| Q63ZK1 | Coiled-coil-helix-coiled-coil-helix domain containing 4 | *Xenopus laevis* |
| Q8K2Q5 | Coiled-coil-helix-coiled-coil-helix domain containing 7 | *Mus musculus* |
| O35796 | Complement component 1, q subcomponent binding protein | *Rattus norvegicus* |
| P50613 | Cyclin-dependent kinase 7 | *Homo sapiens* |
| P10606 | Cytochrome c oxidase subunit Vb | *Homo sapiens* |
| P56391 | Cytochrome c oxidase, subunit vib polypeptide 1 | *Mus musculus* |
| Q8WNV7 | Dehydrogenase/reductase (SDR family) member 4 | *Sus scrofa* |
| Q6AY55 | Dephospho-coa kinase domain containing | *Rattus norvegicus* |
| Q80Y81 | Elac homolog 2 (E. Coli) | *Mus musculus* |
| P38117 | Electron-transfer-flavoprotein, beta polypeptide | *Homo sapiens* |
| Q16595 | Frataxin | *Homo sapiens* |
| Q6PBM1 | Glutaredoxin 5 homolog (S. Cerevisiae) | *Danio rerio* |
| Q9Y2Q3 | Glutathione S-transferase kappa 1 | *Homo sapiens* |
| P28799 | Granulin | *Homo sapiens* |
| Q9W6X3 | Heat shock protein 10 | *Oryzias latipes* |
| Q9WU63 | Heme binding protein 2 | *Mus musculus* |
| Q6P963 | Hydroxyacylglutathione hydrolase | *Danio rerio* |
| O02691 | Hydroxysteroid (17-beta) dehydrogenase 10 | *Bos taurus* |
| Q5U4U5 | Hypothetical LOC495431 | *Xenopus laevis* |
| Q28ED6 | Hypothetical LOC496604 | *Xenopus (Silurana) tropicalis* |
| Q5M8Z2 | Hypothetical LOC496649 | *Xenopus (Silurana) tropicalis* |
| Q2M2S5 | Hypothetical LOC768072 | *Bos taurus* |
| Q8R035 | Immature colon carcinoma cluster 1 | *Mus musculus* |
| Q5ZJ74 | Iron-sulfur cluster assembly 1 homolog (S. Cerevisiae) | *Gallus gallus* |
| Q9DCB8 | Iron-sulfur cluster assembly 2 homolog (S. Cerevisiae) | *Mus musculus* |
| Q0VBY0 | Mature T-cell proliferation 1 | *Bos taurus* |
| Q8IVH4 | Methylmalonic aciduria (cobalamin deficiency) cbla type | *Homo sapiens* |
| Q9D273 | Methylmalonic aciduria (cobalamin deficiency) type B homolog (human) | *Mus musculus* |
| Q9D1I5 | Methylmalonyl coa epimerase | *Mus musculus* |
| Q969V5 | Mitochondrial E3 ubiquitin ligase 1 | *Homo sapiens* |
| Q5HZI9 | Mitochondrial carrier triple repeat 1 | *Mus musculus* |
| Q9UDX5 | Mitochondrial protein 18 kda | *Homo sapiens* |
| Q3TBW2 | Mitochondrial ribosomal protein L10 | *Mus musculus* |
| Q9D1P0 | Mitochondrial ribosomal protein L13 | *Mus musculus* |
| Q9D1I6 | Mitochondrial ribosomal protein L14 | *Mus musculus* |
| Q2TBI6 | Mitochondrial ribosomal protein L32 | *Bos taurus* |
| Q9CQP0 | Mitochondrial ribosomal protein L33 | *Mus musculus* |
| Q9DCU6 | Mitochondrial ribosomal protein L4 | *Mus musculus* |
| Q6DJI4 | Mitochondrial ribosomal protein L41 | *Xenopus laevis* |
| Q08DT6 | Mitochondrial ribosomal protein L47 | *Bos taurus* |
| Q9CQ40 | Mitochondrial ribosomal protein L49; similar to mitochondrial ribosomal protein L49 | *Mus musculus* |
| Q96EL3 | Mitochondrial ribosomal protein L53 | *Homo sapiens* |
| Q9VE04 | Mitochondrial ribosomal protein L55 | *Drosophila melanogaster* |
| Q9VFB2 | Mitochondrial ribosomal protein S10 | *Drosophila melanogaster* |
| O35680 | Mitochondrial ribosomal protein S12 | *Mus musculus* |
| Q9CR88 | Mitochondrial ribosomal protein S14; similar to mitochondrial ribosomal protein S14 | *Mus musculus* |
| Q9V6Y3 | Mitochondrial ribosomal protein S16 | *Drosophila melanogaster* |
| Q99N85 | Mitochondrial ribosomal protein S18A | *Mus musculus* |
| Q767K8 | Mitochondrial ribosomal protein S18B | *Sus scrofa* |
| Q8R2L5 | Mitochondrial ribosomal protein S18C | *Mus musculus* |
| P82920 | Mitochondrial ribosomal protein S21 | *Bos taurus* |
| Q9D125 | Mitochondrial ribosomal protein S25 | *Mus musculus* |
| Q9CY16 | Mitochondrial ribosomal protein S28 | *Mus musculus* |
| Q9VZD5 | Mitochondrial ribosomal protein S6 | *Drosophila melanogaster* |
| Q9CR24 | Nudix (nucleoside diphosphate linked moiety X)-type motif 8 | *Mus musculus* |
| Q9HBH1 | Peptide deformylase (mitochondrial); component of oligomeric golgi complex 8 | *Homo sapiens* |
| Q9Y3E5 | Peptidyl-trna hydrolase 2 | *Homo sapiens* |
| Q07066 | Peroxisomal membrane protein 2 | *Rattus norvegicus* |
| Q61907 | Phosphatidylethanolamine N-methyltransferase | *Mus musculus* |
| O35972 | Predicted gene 13671; mitochondrial ribosomal protein L23 | *Mus musculus* |
| Q9CQL5 | Predicted gene 13675; mitochondrial ribosomal protein L18 | *Mus musculus* |
| O55003 | Predicted gene 14506; BCL2/adenovirus E1B interacting protein 3; predicted gene 6532; similar to E1B 19K/Bcl-2-binding protein homolog | *Mus musculus* |
| Q9D7J4 | Predicted gene 15683; RIKEN cdna 2310005N03 gene; similar to RIKEN cdna 2310005N03 | *Mus musculus* |
| Q9EP80 | Protein interacting with PRKCA 1 | *Rattus norvegicus* |
| Q15119 | Pyruvate dehydrogenase kinase, isozyme 2 | *Homo sapiens* |
| O46504 | Pyruvate dehydrogenase phosphatase regulatory subunit | *Bos taurus* |
| P31399 | Similar to ATP synthase D chain, mitochondrial; ATP synthase, H+ transporting, mitochondrial F0 complex, subunit d | *Rattus norvegicus* |
| Q3MIE0 | Similar to enoyl Coenzyme A hydratase domain containing 3 | *Rattus norvegicus* |
| P21571 | Similar to mitochondrial ATP synthase coupling factor 6; ATP synthase, H+ transporting, mitochondrial F0 complex, subunit F6 | *Rattus norvegicus* |
| Q767K8 | Similar to mitochondrial ribosomal protein S18-2 | *Sus scrofa* |
| Q767K8 | Similar to mitochondrial ribosomal protein S18-2 | *Sus scrofa* |
| Q9UBX3 | Solute carrier family 25 (mitochondrial carrier; dicarboxylate transporter), member 10 | *Homo sapiens* |
| Q08DK7 | Solute carrier family 25, member 29 | *Bos taurus* |
| Q8BGF9 | Solute carrier family 25, member 44 | *Mus musculus* |
| Q9Y6N5 | Sulfide quinone reductase-like (yeast) | *Homo sapiens* |
| Q9WVJ4 | Synaptojanin 2 binding protein | *Rattus norvegicus* |
| Q95108 | Thioredoxin 2 | *Bos taurus* |
| O95881 | Thioredoxin domain containing 12 (endoplasmic reticulum) | *Homo sapiens* |
| Q32LD4 | Transcription factor B2, mitochondrial | *Bos taurus* |
| P62074 | Translocase of inner mitochondrial membrane 10 homolog (yeast) | *Rattus norvegicus* |
| Q9WV98 | Translocase of inner mitochondrial membrane 9 homolog (yeast) | *Mus musculus* |
| Q5RA31 | Translocase of outer mitochondrial membrane 20 homolog (yeast) | *Pongo abelii* |
| Q9P0U1 | Translocase of outer mitochondrial membrane 7 homolog (yeast) | *Homo sapiens* |
| P50637 | Translocator protein | *Mus musculus* |
| Q9CQN6 | Transmembrane protein 14C | *Mus musculus* |
| P00129 | Ubiquinol-cytochrome c reductase binding protein | *Bos taurus* |
| Q9UDW1 | Ubiquinol-cytochrome c reductase complex (7.2 kd) | *Homo sapiens* |
| Q5ZLR5 | Ubiquinol-cytochrome c reductase, Rieske iron-sulfur polypeptide-like 1 | *Gallus gallus* |
| P40337 | Von Hippel-Lindau tumor suppressor | *Homo sapiens* |
| Q499R4 | Yrdc domain containing (E.coli) | *Rattus norvegicus* |
| A3KP37 | Zgc:162919 | *Danio rerio* |
| Q6DGJ3 | Zgc:92895 | *Danio rerio* |
| Q8BGC4 | Zinc binding alcohol dehydrogenase, domain containing 2 | *Mus musculus* |
| Q5ZJ74 | Zinc finger, CCHC domain containing 6 | *Gallus gallus* |

Table S18 Down-regulated *A. digitifera* transcripts likely involved in suppression of the host immune response in *Chromera*-infected larvae at 48 h post-infection with corrected *P* ≤ 0.05. Columns shown are coral cluster ID, annotated protein ID and name, species, E-value and the log_2_fold change values

| Cluster ID | UniProt ID/ gene model ID | Protein Name | Species | E-value | logFC |
| --- | --- | --- | --- | --- | --- |
| adi_EST_assem_5384 | P01027 | Complement C3 | *Mus musculus* | 1.66E-19 | -5.12 |
| adi_EST_assem_15402 | P13671 | Complement component C6 precursor | *Homo sapiens* | 8.95E-50 | -4.84 |
| adi_EST_assem_31512 | Q91132 | Cobra venom factor \| Complement C3 homolog | *Naja kaouthia* | 5.20E-28 | -4.10 |
| adi_EST_assem_14742 | Q9NR16 | Scavenger receptor cysteine-rich type 1 protein M160 | *Homo sapiens* | 6.00E-43 | -4.47 |
| adi_EST_assem_22790 | P30205 | CD163 molecule-like 1 Speract/scavenger receptor | *Bos taurus* | 4.98E-12 | -1.28 |
| adi_EST_assem_8256 | P55259 | glycoprotein 2 (zymogen granule membrane) | *Homo sapiens* | 2.48E-15 | -4.18 |
| adi_EST_assem_4678 | Q14118 | dystroglycan 1 (dystrophin-associated glycoprotein 1) | *Homo sapiens* | 2.89E-27 | -2.37 |
| adi_EST_assem_10380 | Q9TSZ6 | dystroglycan 1 (dystrophin-associated glycoprotein 1) | *Canis lupus* | 7.30E-18 | -2.81 |
| adi_EST_assem_14075 | aug_v2a.04319 | toll-like receptor | *Acropora digitifera* | 3.10E-26 | -1.80 |
| adi_EST_assem_5555 | aug_v2a.02686 | toll-like receptor | *Acropora digitifera* | 3.20E-24 | -1.60 |
| adi_EST_assem_23391 | aug_v2a.03526 | C-type lectin domain family 4 member g (clc4g) | *Acropora digitifera* | 3.20E-17 | -3.70 |
| adi_EST_assem_9676 | Q64449 | C-type mannose receptor 2 (MRC2) | *Mus musculus* | 5.62E-18 | -1.84 |
| adi_EST_assem_13491 | P19838 | nuclear factor of kappa light polypeptide gene enhancer in B-cells 1 | *Homo sapiens* | 2E-167 | -1.28 |
| adi_EST_assem_8683 | Q8K3Z0 | nucleotide-binding oligomerization domain containing 2 | *Mus musculus* | 2.32E-16 | -2.00 |
| adi_EST_assem_461 | Q9BUZ4 | TNF receptor-associated factor 4 | *Homo sapiens* | 2.44E-84 | -1.63 |
| adi_EST_assem_3655 | P21580 | tumor necrosis factor, alpha-induced protein 3 | *Homo sapiens* | 1.00E-64 | -1.20 |
| adi_EST_assem_9748 | O95163 | inhibitor of kappa light polypeptide gene enhancer in B-cells, kinase complex-associated protein | *Homo sapiens* | 9.26E-94 | -3.50 |
| adi_EST_assem_6149 | Q9ESE1 | LPS-responsive beige-like anchor | *Mus musculus* | 0 | -2.38 |
| adi_EST_assem_18500 | Q91880 | suppressor of hairless protein 1 | *Xenopus laevis* | 3.01E-40 | -3.19 |
| adi_EST_assem_10795 | Q8N8V2 | guanylate binding protein 7 | *Homo sapiens* | 3.46E-42 | -2.48 |
| adi_EST_assem_4061 | P36888 | fms-related tyrosine kinase 3\| CD_antigen: CD135 | *Homo sapiens* | 4.7E-42 | -2.37 |
| adi_EST_assem_10633 | Q9NW08 | polymerase (RNA) III (DNA directed) polypeptide B | *Homo sapiens* | 1.13E-156 | -2.67 |
| adi_EST_assem_8717 | O14802 | polymerase (RNA) III (DNA directed) polypeptide A, 155kDa | *Homo sapiens* | 0 | -2.50 |
| adi_EST_assem_3715 | P80025 | perl_lactoperoxidase | *Bos taurus* | 1.22E-12 | -2.54 |
| adi_EST_assem_5779 | Q9JJ22 | endoplasmic reticulum aminopeptidase 1 | *Rattus norvegicus* | 3.66E-43 | -3.33 |
| adi_EST_assem_9934 | Q96JA1 | leucine-rich repeats and immunoglobulin-like domains 1 | *Homo sapiens* | 7.72E-21 | -5.19 |
| adi_EST_assem_28915 | Q9DE07 | nibrin | *Gallus gallus* | 9.80E-23 | -4.66 |
| adi_EST_assem_17218 | P16621 | Leukocyte-antigen-related-like | *Drosophila melanogaster* | 1.81E-26 | -2.79 |
| adi_EST_assem_2273 | O18738 | dystroglycan 1 (dystrophin-associated glycoprotein 1) | *Bos taurus* | 3.44E-21 | -2.33 |
| adi_EST_assem_858 | Q95218 | Dmbt1 deleted in malignant brain tumors 1 | *Oryctolagus cuniculus* | 5.64E-112 | -2.32 |
| adi_EST_assem_26531 | Q8N6G6 | ADAMTS-like 1 | *Homo sapiens* | 2.31E-25 | -2.31 |
| adi_EST_assem_2070 | Q3UG20 | myeloid/lymphoid or mixed-lineage leukemia 5\| Histone-lysine N-methyltransferase 2E | *Mus musculus* | 1.22E-71 | -2.26 |
| adi_EST_assem_3633 | Q9UGM3 | deleted in malignant brain tumors 1\| Glycoprotein 340 | *Homo sapiens* | 4.32E-81 | -2.25 |
| adi_EST_assem_2961 | Q05BQ1 | immunoglobulin superfamily, member 9 | *Mus musculus* | 1.10E-48 | -2.14 |
| adi_EST_assem_8762 | P58022 | lysyl oxidase-like 2 | *Mus musculus* | 8.67E-75 | -2.14 |
| adi_EST_assem_683 | Q4VGL6 | RING CCCH (C3H) domains 1\| Roquin-1 | *Mus musculus* | 5.58E-173 | -2.01 |
| adi_EST_assem_4797 | Q8WWQ8 | stabilin 2 | *Homo sapiens* | 9.75E-145 | -2.01 |
| adi_EST_assem_638 | Q10741 | ADAM metallopeptidase domain 10 | *Bos taurus* | 1.23E-161 | -1.99 |
| adi_EST_assem_7004 | B4F6N6 | lysyl oxidase-like 2 | *Xenopus (Silurana) tropicalis* | 3.04E-100 | -1.94 |
| adi_EST_assem_5267 | P46531 | Notch homolog 1, translocation-associated (Drosophila) | *Homo sapiens* | 7.82E-32 | -1.84 |
| adi_EST_assem_3259 | Q62077 | phospholipase C, gamma 1 | *Mus musculus* | 0 | -1.70 |
| adi_EST_assem_4854 | Q05B92 | transcription factor binding to IGHM enhancer 3 | *Bos taurus* | 4.23E-54 | -1.65 |
| adi_EST_assem_4981 | P31266 | recombination signal binding protein for immunoglobulin kappa J region | *Mus musculus* | 0 | -1.62 |
| adi_EST_assem_7676 | Q91035 | sonic hedgehog homolog (Drosophila) | *Gallus gallus* | 6.31E-97 | -1.59 |
| adi_EST_assem_26435 | O13076 | adenosine A2b receptor | *Gallus gallus* | 1.10E-17 | -1.50 |
| adi_EST_assem_580 | Q9UHD2 | TANK-binding kinase 1 (TBK1) | *Homo sapiens* | 3.05E-144 | -1.45 |
| adi_EST_assem_3427 | A6H7G2 | drebrin-like | *Bos taurus* | 1.26E-43 | -1.32 |
| adi_EST_assem_1987 | P42232 | signal transducer and activator of transcription 5B | *Mus musculus* | 6.98E-108 | -1.31 |
| adi_EST_assem_5314 | Q5R9T9 | guanylate binding protein family, member 6 | *Pongo abelii* | 1.29E-49 | -1.31 |
| adi_EST_assem_1695 | Q7ZW34 | zgc:55318 \| Contactin-5 | *Danio rerio* | 2.59E-18 | -1.23 |
| adi_EST_assem_1190 | O54928 | suppressor of cytokine signaling 5\| Cytokine-inducible SH2-containing protein 5 | *Mus musculus* | 5.93E-57 | -1.20 |
| adi_EST_assem_3877 | Q9PVW8 | sal_silasrhamnose-binding lectin | *Silurus asotus* | 1.62E-08 | -1.54 |
| adi_EST_assem_1432 | Q66S03 | lecg_galactose-specific lectin nattectin | *Thalassophryne nattereri* | 1.49E-12 | -1.98 |
| adi_EST_assem_1463 | Q0V8S9 | cntp5_contactin-associated 5 | *gallus gallus* | 7.24E-14 | -1.90 |
| adi_EST_assem_35905 | P17336 | cata_catalase | *Drosophila melanogaster* | 6.04E-70 | -2.44 |
| adi_EST_assem_3532 | aug_v2a.12354 | SODC_Superoxide dismutase [Cu-Zn] | *Acropora digitifera* | 4.70E-38 | -2.24 |
| adi_EST_assem_3531 | aug_v2a.01713 | SODC1_Superoxide dismutase [Cu-Zn] 1 | *Acropora digitifera* | 0.00E+00 | -1.26 |
| adi_EST_assem_4182 | P12527 | lox5_arachidonate 5-lipoxygenase | *Rattus norvegicus* | 4.90E-46 | -1.04 |
| adi_EST_assem_2688 | P48999 | lox5_arachidonate 5-lipoxygenase | *Mus musculus* | 1.35E-104 | -1.47 |

Table S19 Differential expression of *A. digitifera* transcripts likely involved in phagocytosis in *Chromera*-infected larvae at 48 h post infection with corrected *P* ≤ 0.05. Columns shown are coral cluster ID, annotated protein ID and name, species, E-value and the log_2_fold change values

| Cluster ID | Protein ID | Protein name | Species | E-value | Log FC |
| --- | --- | --- | --- | --- | --- |
| adi_EST_assem_14553 | Q5SV85 | AP1 gamma subunit binding protein 1 | *Mus musculus* | 3.02E-17 | -3.88 |
| adi_EST_assem_5204 | Q9NZM3 | Intersectin 2 | *Homo sapiens* | 0 | -2.50 |
| adi_EST_assem_6352 | Q9WV76 | Adaptor-related protein complex AP-4, beta 1 | *Mus musculus* | 1.14E-145 | -2.28 |
| adi_EST_assem_286 | P18484 | Adaptor-related protein complex 2, alpha 2 subunit | *Rattus norvegicus* | 0 | -2.04 |
| adi_EST_assem_6885 | P98155 | Very low density lipoprotein receptor | *Homo sapiens* | 8.13E-162 | -1.81 |
| adi_EST_assem_4258 | P22892 | Adaptor protein complex AP-1, gamma 1 subunit | *Mus musculus* | 0 | -1.79 |
| adi_EST_assem_14154 | Q8WXE9 | Stonin 2 | *Homo sapiens* | 3.65E-62 | -1.74 |
| adi_EST_assem_918 | Q9I8D1 | Myosin VI | *Gallus gallus* | 0 | -1.50 |
| adi_EST_assem_4580 | Q14677 | Clathrin interactor 1 | *Homo sapiens* | 3.26E-82 | -1.49 |
| adi_EST_assem_749 | P98156 | Very low density lipoprotein receptor | *Mus musculus* | 6.74E-155 | -1.37 |
| adi_EST_assem_2285 | O08585 | Clathrin, light polypeptide (Lca) | *Mus musculus* | 2.63E-34 | 1.08 |

Table S20 Differential expression of *A. digitifera* transcripts likely involved in early and/or late endosome formation and phagosomal maturation in *Chromera*-infected larvae at 48 h post infection with corrected *P* ≤ 0.05. Columns shown are coral cluster ID, annotated protein ID and name, species, E-value and the log_2_fold change values

| Cluster ID | UniProt/gene model ID | Protein name | Species | E value | logFC |
| --- | --- | --- | --- | --- | --- |
| adi_EST_assem_12994 | aug_v2a.14153 | Zinc finger FYVE domain-containing protein, early endosome antigen 1 (EEA1) | *Acropora digitifera* | 5.00E-18 | -4.11 |
| adi_EST_assem_13736 | aug_v2a.14153 | Zinc finger FYVE domain-containing protein, early endosome antigen 1 (EEA1) | *Acropora digitifera* | 5.00E-18 | -3.50 |
| adi_EST_assem_24995 | Q96Q42 | Amyotrophic lateral sclerosis 2 (juvenile) | *Homo sapiens* | 1.76E-14 | -3.19 |
| adi_EST_assem_21892 | Q920R0 | Amyotrophic lateral sclerosis 2 (juvenile) homolog (human) | *Mus musculus* | 1.20E-29 | -5.53 |
| adi_EST_assem_12817 | P0C5Y8 | Amyotrophic lateral sclerosis 2 (juvenile) homolog (human) | *Rattus norvegicus* | 0 | -1.53 |
| adi_EST_assem_13263 | P42356 | Phosphatidylinositol 4-kinase, catalytic, alpha PI4KA | *Homo sapiens* | 4.25E-63 | -2.35 |
| adi_EST_assem_23765 | O08662 | Phosphatidylinositol 4-kinase, catalytic, alpha PI4KA | *Rattus norvegicus* | 7.42E-93 | -2.40 |
| adi_EST_assem_8096 | A9X1A0 | Phosphatidylinositol 4-kinase, catalytic, beta PI4KB | *Papio anubis* | 0 | -1.69 |
| adi_EST_assem_6472 | O00750 | Phosphoinositide-3-kinase, class 2, beta polypeptide PIK3C2B | *Homo sapiens* | 2.83E-25 | -5.20 |
| adi_EST_assem_25124 | O88763 | Phosphoinositide-3-kinase, class 3  PIK3C3 | *Rattus norvegicus* | 3.98E-164 | -3.58 |
| adi_EST_assem_5459 | Q9WVR3 | Inositol polyphosphate phosphatase-like 1 INPPL1 | *Rattus norvegicus* | 8.17E-169 | -1.17 |
| adi_EST_assem_6577 | Q92609 | TBC1 domain family, member 5  TBC1D5 | *Homo sapiens* | 2.43E-130 | -1.67 |
| adi_EST_assem_5136 | P51149 | Ras-related protein Rab-7a | *Homo sapiens* | 4.00E-33 | 1.80 |
| adi_EST_assem_10699 | P28648 | CD63 molecule \| Mast cell antigen AD1 | *Rattus norvegicus* | 3.38E-25 | 1.38 |
| adi_EST_assem_12482 | Q5ZL74 | Vesicle-associated membrane protein 7 (VAMP7) | *Gallus gallus)* | 3.61E-87 | 1.34 |
| adi_EST_assem_5509 | aug_v2a.01938 | Lysosomal-associated membrane protein 1\| LAMP1 | *Acropora digitifera* | 1.70E-24 | 1.31 |
| adi_EST_assem_3641 | P50408 | Atpase, H transporting, lysosomal V1 subunit F | *Rattus norvegicus* | 2.00E-54 | 1.22 |
| adi_EST_assem_4901 | Q2TA24 | Atpase, H+ transporting, lysosomal 21kda, V0 subunit b | *Bos taurus* | 9.55E-70 | 1.00 |
| adi_EST_assem_9805 | Q9SIQ9 | Vesicle-associated membrane protein 712 (VAMP712) | *Arabidopsis thaliana* | 1.33E-44 | 1.05 |
| adi_EST_assem_32654 | Q9P6K1 | Protein transport protein sft2 | *Schizosaccharomyces pombe* | 4.10E-18 | 1.25 |
| adi_EST_assem_15506 | O95295 | SNAP-associated protein  SNAPIN | *Homo sapiens* | 4.71E-36 | 1.37 |
| adi_EST_assem_8353 | Q7ZXB7 | Late endosomal/lysosomal adaptor and MAPK and MTOR activator 2-B  lamtor2-b | *Xenopus laevis* | 4.34E-54 | 1.59 |

Table S21 Differential expression of *A. digitifera* transcripts likely involved in autophagy and lysosome functions in *Chromera*-infected larvae at 48 h post infection with corrected *P* ≤ 0.05. Columns shown are coral cluster ID, annotated protein ID and name, species, E-value and the log_2_fold change values

| Cluster ID | UniProt ID | Protein name | E-value | logFC |
| --- | --- | --- | --- | --- |
| adi_EST_assem_2028 | P60517 | GABA(A) receptor-associated protein Rattus norvegicus | 1.36E-75 | 1.21 |
| adi_EST_assem_24964 | Q2TBJ5 | ATG12 autophagy related 12 homolog (S. cerevisiae) Rattus norvegicus | 2.44E-32 | 1.57 |
| adi_EST_assem_4839 | Q9CPX6 | autophagy-related 3 (yeast) Mus musculus | 1.32E-140 | 1.14 |
| adi_EST_assem_22677 | Q99J83 | autophagy-related 5 (yeast) Mus musculus | 1.07E-116 | 1.77 |
| adi_EST_assem_4610 | Q2HJ23 | microtubule-associated protein 1 light chain 3 alpha Bos taurus | 1.03E-46 | 1.17 |
| adi_EST_assem_17647 | Q6PCJ9 | Lysosomal thioesterase PPT2-A Xenopus laevis | 2.31E-93 | 1.14 |
| adi_EST_assem_8111 | P48441 | iduronidase, alpha-L- Mus musculus | 7.85E-27 | 1.19 |
| adi_EST_assem_1447 | Q86YJ5 | E3 ubiquitin-protein ligase MARCH9 Homo sapiens Homo sapiens | 6.34E-23 | 1.35 |
| adi_EST_assem_1823 | P38571 | lipase A, lysosomal acid, cholesterol esterase Homo sapiens | 1.21E-31 | -1.79 |
| adi_EST_assem_14969 | Q29444 | mannosidase, beta A, lysosomal Bos taurus | 2.62E-163 | -1.66 |
| adi_EST_assem_25764 | O00462 | mannosidase, beta A, lysosomal Homo sapiens | 1.35E-31 | -6.45 |

Table S22 Differential expression of *A. digitifera* transcripts likely involved in endosomal trafficking in *Chromera*-infected larvae at 48 h post infection with corrected *P* ≤ 0.05. Columns shown are coral cluster ID, annotated protein ID and name, species, E-value and the log_2_fold change values

| Cluster ID | UniProt ID | Protein name | E value | logFC |
| --- | --- | --- | --- | --- |
| adi_EST_assem_16252 | P59015 | Vacuolar protein sorting protein 18 Danio rerio | 3.53E-87 | -5.11 |
| adi_EST_assem_4366 | Q91W86 | Vacuolar protein sorting 11 (yeast) Mus musculus | 0 | -2.26 |
| adi_EST_assem_15180 | Q920Q4 | Vacuolar protein sorting 16 (yeast) Mus musculus | 0 | -1.94 |
| adi_EST_assem_11058 | Q8R5L3 | Vacuolar protein sorting 39 (yeast) Mus musculus | 0 | -1.86 |
| adi_EST_assem_3691 | Q8R0H9 | Golgi associated, gamma adaptin ear containing, ARF binding protein 1 Mus musculus | 1.86E-87 | -1.33 |
| adi_EST_assem_7197 | A2RSQ0 | DENN/MADD domain containing 5B Mus musculus | 0 | -1.53 |
| adi_EST_assem_2060 | Q5ZJ17 | RAB gtpase activating protein 1-like Gallus gallus | 1.14E-169 | -2.04 |
| adi_EST_assem_8022 | O75154 | RAB11 family interacting protein 3 (class II) Homo sapiens | 9.14E-29 | -2.14 |
| adi_EST_assem_12934 | Q15042 | RAB3 gtpase activating protein subunit 1 (catalytic) Homo sapiens | 5.64E-164 | -1.99 |
| adi_EST_assem_528 | Q8BMG7 | RAB3 gtpase activating protein subunit 2 Mus musculus | 1.47E-74 | -1.96 |
| adi_EST_assem_13048 | Q9H2M9 | RAB3 gtpase activating protein subunit 2 (non-catalytic) Homo sapiens | 0 | -3.46 |
| adi_EST_assem_3833 | Q62739 | RAB3A interacting protein (rabin3) Rattus norvegicus | 3.67E-64 | -2.24 |
| adi_EST_assem_948 | P47736 | RAP1 gtpase activating protein Homo sapiens | 8.98E-113 | -1.22 |
| adi_EST_assem_9191 | Q8IV61 | RAS guanyl releasing protein 3 (calcium and DAG-regulated) Homo sapiens | 3.81E-134 | -1.36 |
| adi_EST_assem_6077 | P28818 | RAS protein-specific guanine nucleotide-releasing factor 1 Rattus norvegicus | 4.11E-15 | -2.89 |
| adi_EST_assem_22365 | Q5F361 | TBC domain-containing protein kinase-like Gallus gallus | 0 | -1.05 |
| adi_EST_assem_1683 | Q86TI0 | TBC1 (tre-2/USP6, BUB2, cdc16) domain family, member 1 Homo sapiens | 1.57E-117 | -2.28 |
| adi_EST_assem_23011 | Q8C9V1 | TBC1 domain family, member 10c Mus musculus | 2.71E-34 | -2.66 |
| adi_EST_assem_1755 | A6H7I8 | TBC1 domain family, member 14 Bos taurus | 1.45E-169 | -1.11 |
| adi_EST_assem_5379 | Q9D9I4 | TBC1 domain family, member 20 Mus musculus | 1.63E-99 | -1.45 |
| adi_EST_assem_4543 | Q9NU19 | TBC1 domain family, member 22B Homo sapiens | 1.38E-162 | -1.25 |
| adi_EST_assem_20484 | Q9UPU7 | TBC1 domain family, member 2B Homo sapiens | 2.50E-37 | -2.25 |
| adi_EST_assem_26789 | Q0VCJ7 | RAS-like, estrogen-regulated, growth inhibitor Bos taurus (Bos taurus) | 1.41E-15 | 2.04 |
| adi_EST_assem_1935 | P22125 | Ras-related protein ORAB-1 Discopyge ommata (Discopyge ommata) | 1.86E-89 | 1.89 |
| adi_EST_assem_1446 | Q05975 | Ras-related protein Rab-2 Lymnaea stagnalis (Lymnaea stagnalis) | 3.00E-136 | 1.62 |
| adi_EST_assem_1323 | Q9UI14 | Rab acceptor 1 (prenylated) Homo sapiens (Homo sapiens) | 5.37E-29 | 1.48 |
| adi_EST_assem_1748 | Q6DHC1 | RAB18B, member RAS oncogene family Danio rerio (Danio rerio) | 1.19E-108 | 1.28 |
| adi_EST_assem_17049 | Q99P75 | RAB9A, member RAS oncogene family Rattus norvegicus (Rattus norvegicus) | 1.34E-69 | 1.23 |
| adi_EST_assem_11824 | O95755 | RAB36, member RAS oncogene family Homo sapiens (Homo sapiens) | 7.16E-79 | 1.08 |
| adi_EST_assem_12773 | Q5RAV6 | RAB6A, member RAS oncogene family Pongo abelii Pongo abelii | 2.03E-123 | 1.05 |
| adi_EST_assem_452 | Q5R5U1 | RAB10, member RAS oncogene family Pongo abelii Pongo abelii | 2.79E-106 | 1.00 |
| adi_EST_assem_6154 | A4IHM6 | Rab-like protein 3 Xenopus (Silurana) tropicalis | 6.18E-69 | 1.81 |
| adi_EST_assem_12084 | Q5RFI2 | RAB28, member RAS oncogene family Pongo abelii | 6.18E-93 | 1.76 |
| adi_EST_assem_20522 | Q5M7D1 | Rab and dnaj domain-containing protein B Xenopus laevis | 1.70E-26 | 1.71 |
| adi_EST_assem_11749 | Q6IMK3 | Rab and dnaj domain-containing protein Danio rerio | 1.60E-07 | 1.35 |
| adi_EST_assem_22058 | Q0VCN3 | RAB, member of RAS oncogene family-like 4 Bos taurus | 2.16E-66 | 1.30 |
| adi_EST_assem_4227 | P25228 | Rab-protein 3 Drosophila melanogaster | 3.70E-131 | 1.13 |

Table S23 Differential expression of *A. digitifera* transcripts likely to have pro-apoptotic functions in *Chromera*-infected larvae at 48 h post infection with corrected *P* ≤ 0.05. Columns shown are coral cluster ID, annotated protein ID and name, species, E-value and the log_2_fold change values

| Cluster ID | Protein ID | Protein Name | Species | E-value | LogFC |  |
| --- | --- | --- | --- | --- | --- | --- |
| adi_EST_assem_13903 | aug_v2a.09191 | Death-domain containing protein (CRADD) | Acropora digitifera | 1.10E-13 | -1.20 |  |
| adi_EST_assem_2563 | Q6Q0C0 | TNF receptor-associated factor 7 | Homo sapiens | 0.00E+00 | -2.44 |  |
| adi_EST_assem_756 | Q96B97 | SH3-domain kinase binding protein 1 | Homo sapiens | 3.34273E-25 | -1.41 |  |
| adi_EST_assem_9504 | Q5ZM55 | fem-1 homolog b (C. elegans) | Gallus gallus | 6.1464E-151 | -2.11 |  |
| adi_EST_assem_8567 | Q80U28 | MAP-kinase activating death domain | Mus musculus | 3.963E-164 | -2.06 |  |
| adi_EST_assem_17887 | O14727 | apoptotic peptidase activating factor 1 (APAF1) | Homo sapiens | 2.30E-56 | -2.39 |  |
| adi_EST_assem_15181 | Q9WTU6 | mitogen-activated protein kinase 9 | Mus musculus | 2.27E-40 | -1.87 |  |
| adi_EST_assem_2238 | Q6GQJ2 | MGC79115 protein \| Protein Jade-1 | Xenopus laevis | 6.85E-138 | -1.12 |  |
| adi_EST_assem_3843 | Q9UKV3 | apoptotic chromatin condensation inducer 1 | Homo sapiens | 4.03E-28 | -1.65 |  |
| adi_EST_assem_10928 | Q96EY1 | DnaJ (Hsp40) homolog, subfamily A, member 3 | Homo sapiens | 8.47E-128 | -1.81 |  |
| adi_EST_assem_3208 | Q9H2X6 | homeodomain interacting protein kinase 2 | Homo sapiens | 0.00E+00 | -1.56 |  |
| adi_EST_assem_14775 | [P50591](http://www.uniprot.org/uniprot/P50591) | Tumor necrosis factor ligand superfamily member 10 (TNF-related apoptosis inducing ligand) | Homo sapiens | 4.12E-11 | 1.09 |  |
| adi_EST_assem_6756 | Q12983 | BCL2/adenovirus E1B 19 kDa protein-interacting protein 3 | Homo sapiens | 1.17E-38 | 1.26 |  |
| adi_EST_assem_20578 | Q9UDX5 | mitochondrial protein 18 kDa | Homo sapiens | 1.59E-18 | 2 |  |
| adi_EST_assem_17828 | Q95KV7 | NADH dehydrogenase (ubiquinone) 1 alpha subcomplex, 13 | Bos taurus | 7.94E-38 | 1.19 |  |
| adi_EST_assem_11324 | P51397 | death-associated protein 1 | Homo sapiens | 1.21E-17 | 1.78 |  |
| adi_EST_assem_18148 | O14681 | p53-induced gene 8 protein | Homo sapiens | 4.41E-33 | 1.16 |  |
| adi_EST_assem_22477 | Q6DJI4 | mitochondrial ribosomal protein L41 | Xenopus laevis | 5.45E-15 | 1.17 |  |
| adi_EST_assem_22700 | Q9Y3E5 | peptidyl-tRNA hydrolase 2 | Homo sapiens | 1.93E-56 | 1.84 |  |
| adi_EST_assem_9885 | A0AUR5 | Protein FAM188A | Danio rerio | 5.95E-48 | 1.09 |  |
| adi_EST_assem_20423 | Q969V5 | mitochondrial E3 ubiquitin ligase 1 | Homo sapiens | 1.30E-64 | 1.27 |  |
| adi_EST_assem_3403 | P12815 | programmed cell death 6 | Mus musculus | 3.39E-76 | 1.27 |  |

Table S24 Differential expression of *A. digitifera* transcripts likely to have anti-apoptotic functions in *Chromera*-infected larvae at 48 h post infection with corrected *P* ≤ 0.05. Columns shown are coral cluster ID, annotated protein ID and name, species, E-value and the log_2_fold change values

| Cluster ID | Protein ID | | | Protein Name | Species | | E-value | | logFC | |  |  |
| --- | --- | --- | --- | --- | --- | --- | --- | --- | --- | --- | --- | --- |
| adi_EST_assem_18830 | | Q9ER63 | Tumor necrosis factor receptor superfamily member 23 | | | *Mus musculus* | | 4.19E-21 | | -2.73 |  |  |
| adi_EST_assem_4232 | | Q8K4J6 | MKL (megakaryoblastic leukemia)/myocardin-like 1 | | | *Mus musculus* | | 7.27E-26 | | -1.16 |  |  |
| adi_EST_assem_21510 | | O08863 | baculoviral IAP repeat-containing 3 | | | *Mus musculus* | | 9.79E-30 | | -2.13 |  |  |
| adi_EST_assem_14845 | | P56597 | Inhibitor of p53-induced apoptosis-beta | | | *Homo sapiens* | | 9.34E-13 | | -5.41 |  |  |
| adi_EST_assem_8731 | | O95429 | BCL2-associated athanogene 4 \| Silencer of death domains | | | *Homo sapiens* | | 4.28E-13 | | -1.29 |  |  |
| adi_EST_assem_4215 | | O88738 | baculoviral IAP repeat-containing 6 | | | *Mus musculus* | | 1.40E-22 | | -2.88 |  |  |
| adi_EST_assem_6529 | | Q9NR09 | baculoviral IAP repeat-containing 6 | | | *Homo sapiens* | | 4.00E-179 | | -1.23 |  |  |
| adi_EST_assem_3312 | | Q6P132 | Tax1 (human T-cell leukemia virus type I) binding protein 1b | | | *Danio rerio* | | 2.02E-37 | | -2.05 |  |  |
| adi_EST_assem_12506 | | Q4G017 | nischarin | | | *Rattus norvegicus* | | 6.03E-23 | | -1.26 |  |  |
| adi_EST_assem_2057 | | Q8CHN6 | sphingosine-1-phosphate lyase 1 | | | *Rattus norvegicus* | | 0 | | -1.02 |  |  |
| adi_EST_assem_18171 | | Q12933 | TNF receptor-associated factor 2 | | | *Homo sapiens* | | 8.64E-11 | | 1.60 |  |  |
| adi_EST_assem_24012 | | P70191 | TNF receptor-associated factor 5 | | | *Mus musculus* | | 5.61E-15 | | 1.30 |  |  |
| adi_EST_assem_4585 | | Q00709 | Apoptosis regulator Bcl-2 \| B-cell CLL/lymphoma 2 | | | *Gallus gallus* | | 1.33E-18 | | 1.31 |  |  |
| adi_EST_assem_3951 | | Q99933 | BAG family molecular chaperone regulator 1 | | | *Homo sapiens* | | 2.33E-30 | | 1.25 |  |  |
| adi_EST_assem_2446 | | Q91827 | Apoptosis regulator R1 | | | *Xenopus laevis* | | 2.42E-26 | | 1.55 |  |  |
| adi_EST_assem_8388 | | Q8R5H8 | Fas apoptotic inhibitory molecule | | | *Rattus norvegicus* | | 2.44E-84 | | 1.11 |  | |

Table S25 Differential expression of *A. digitifera* transcripts likely involved in regulation of apoptosis in *Chromera*-infected larvae at 48 h post infection with corrected *P* ≤ 0.05. Columns shown are coral cluster ID, annotated protein ID and name, species, E-value and the log_2_fold change values

| Cluster ID | Protein ID | Protein Name | Species | E-value | logFC |
| --- | --- | --- | --- | --- | --- |
| adi_EST_assem_1890 | Q9P289 | Serine/threonine protein kinase MST4 | *Homo sapiens* | 3.54E-149 | -1.27 |
| adi_EST_assem_5478 | Q923E4 | Sirtuin 1 (silent mating type information regulation 2, homolog) 1 (S. Cerevisiae) | *Mus musculus* | 8.98E-150 | -1.84 |
| adi_EST_assem_8857 | Q0IHU9 | Hypothetical protein MGC145921 | *Xenopus (Silurana) tropicalis* | 1.28E-18 | -1.60 |
| adi_EST_assem_587 | Q5F499 | Optic atrophy 1 (autosomal dominant) | *Gallus gallus* | 0 | -1.96 |
| adi_EST_assem_1205 | Q6NS46 | Programmed cell death 11 | *Mus musculus* | 0 | -1.93 |
| adi_EST_assem_2986 | Q8WUM4 | Programmed cell death 6 interacting protein | *Homo sapiens* | 0 | -1.20 |
| adi_EST_assem_4472 | P21127 | Similar to cell division cycle 2-like 1 (PITSLRE proteins); cell division cycle 2-like 1 (PITSLRE proteins); cell division cycle 2-like 2 (PITSLRE proteins) | *Homo sapiens* | 0 | -1.43 |
| adi_EST_assem_4761 | P05625 | V-raf-1 murine leukemia viral oncogene homolog 1 | *Gallus gallus* | 8.16E-177 | -1.54 |
| adi_EST_assem_11971 | Q9ESK9 | RB1-inducible coiled-coil 1 | *Mus musculus* | 5.39E-49 | -2.48 |
| adi_EST_assem_1233 | Q8N201 | Integrator complex subunit 1 | *Homo sapiens* | 6.27E-116 | -3.67 |
| adi_EST_assem_1941 | Q6P4S8 | Integrator complex subunit 1 | *Mus musculus* | 2.83E-88 | -1.52 |
| adi_EST_assem_4220 | P51111 | Huntingtin | *Rattus norvegicus* | 0 | -1.90 |
| adi_EST_assem_3436 | P00519 | C-abl oncogene 1, receptor tyrosine kinase | *Homo sapiens* | 0 | -1.76 |
| adi_EST_assem_469 | Q5ZIU3 | Dual-specificity tyrosine-(Y)-phosphorylation regulated kinase 2 | *Gallus gallus* | 1.09E-94 | -2.02 |
| adi_EST_assem_6133 | P05696 | Protein kinase C, alpha | *Rattus norvegicus* | 0 | -2.47 |
| adi_EST_assem_4877 | P09215 | Protein kinase C, delta | *Rattus norvegicus* | 0 | -1.35 |
| adi_EST_assem_8760 | Q02156 | Protein kinase C, epsilon | *Homo sapiens* | 0 | -1.51 |
| adi_EST_assem_1629 | Q5PQS4 | GULP, engulfment adaptor PTB domain containing 1 | *Rattus norvegicus* | 2.38E-49 | -1.30 |
| adi_EST_assem_12712 | Q14185 | Dedicator of cytokinesis 1 | *Homo sapiens* | 0 | -2.17 |
| adi_EST_assem_7899 | Q8BUR4 | Dedicator of cytokinesis 1 | *Mus musculus* | 0 | -2.84 |
| adi_EST_assem_4797 | Q8WWQ8 | Stabilin 2 | *Homo sapiens* | 9.75E-145 | -2.02 |
| adi_EST_assem_18171 | Q13114 | TNF receptor-associated factor 3 | *Homo sapiens* | 1.97E-04 | 1.6 |
| adi_EST_assem_24012 | P70191 | TNF receptor-associated factor 5 | *Mus musculus* | 5.61E-15 | 1.3 |
| adi_EST_assem_22957 | Q5JPI3 | Uncharacterized protein c3orf38 | *Homo sapiens* | 6.81E-45 | 1.43 |
| adi_EST_assem_4841 | Q6DF07 | Programmed cell death 10 | *Xenopus tropicalis* | 9.77E-44 | 1.47 |
| adi_EST_assem_7217 | Q2YDC9 | Programmed cell death 2 | *Bos taurus* | 8.88E-39 | 1.3 |
| adi_EST_assem_11984 | Q8BKD6 | E3 ubiquitin-protein ligase RNF144B | *Mus musculus* | 1.92E-73 | 1.43 |
| adi_EST_assem_3525 | Q58CU4 | Probable palmitoyltransferase ZDHHC16 | *Bos taurus* | 1.27E-06 | 2.46 |
| adi_EST_assem_22677 | Q99J83 | Autophagy-related 5 (yeast) | *Mus musculus* | 1.07E-116 | 1.76 |
| adi_EST_assem_17635 | Q8JHF0 | Presenilin enhancer 2 homolog | *Danio rerio* | 6.96E-45 | 1.19 |

**Supplementary Figures**

**
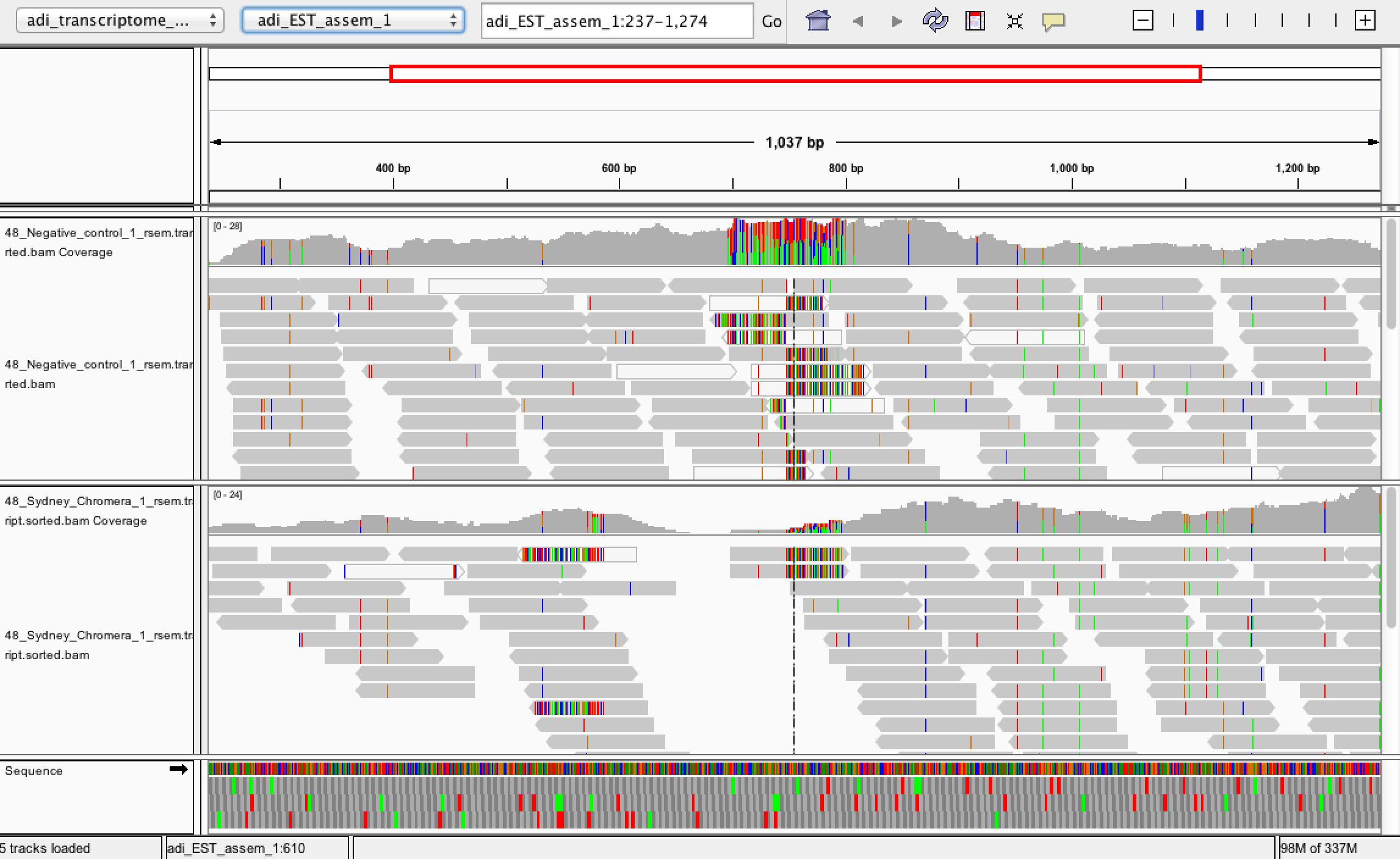
**

Figure S1 A screenshot of *Acropora digitifera* transcripts and read alignments of both control (first block) and *Chromera* infection (second block) samples at the 48 h time point. Using the Integrated Genomics Viewer (IGV), the sorted BAM files containing the aligned reads were uploaded as well as the reference transcriptome sequence data. Illumina reads are represented in gray, while reads with low quality mapping are represented as unshaded (white). The coloured vertical lines show base mismatches to the reference sequence.

Figure S2 Level of agreement amongst the biological replicates at 4 h post *Chromera* infection. The heat map shows the hierarchically clustered Spearman correlation matrix resulting from comparing the transcript expression values (TMM-normalized FPKM) for all samples against one another. Sample clustering indicates the consistency between the biological replicates of the *Chromera* infection (samples I1, I2, I3) and negative control conditions (samples C1, C2, C3) at the 4h time point. The level of correlation is presented by a color field that ranges from green (correlation coefficient 0.8) to red (correlation coefficient 1.0).

Figure S3 Level of agreement amongst the biological replicates at 48 h post *Chromera* infection. The heat map shows the hierarchically clustered Spearman correlation matrix resulting from comparing the transcript expression values (TMM-normalized FPKM) for all samples against one another. Sample clustering indicates the consistency between the biological replicates of the *Chromera* infection (samples I1, I2, I3) and negative control conditions (samples C1, C2, C3) at the 4h time point. The level of correlation is presented by a color field that ranges from green (correlation coefficient 0.75) to red (correlation coefficient 1.0).

Figure S4 A multidimensional scaling (MDS) plot produced by edgeR showing the relationship between all the replicates of *Chromera* infection (samples I1, I2, I3) and negative control conditions (samples C1, C2, C3) at the 48h time point. The distances shown are the biological coefficient of variation (BCV) between samples.

**
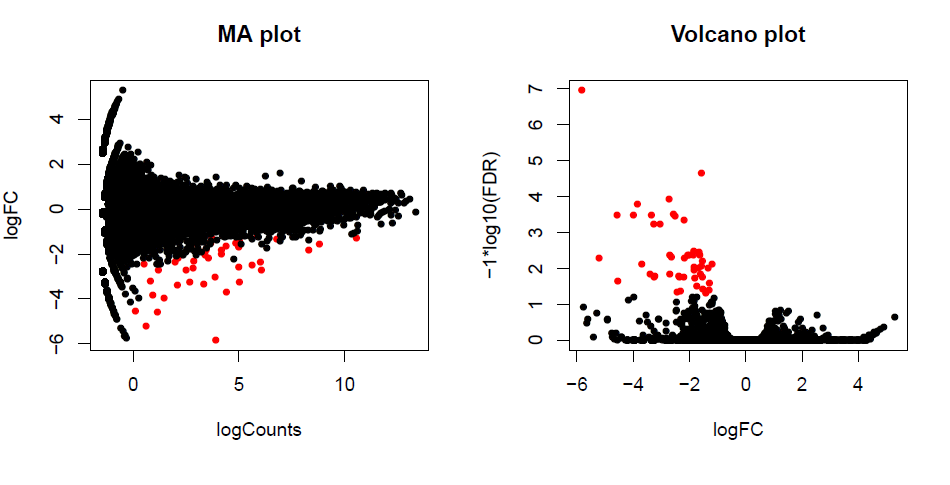
**

Figure S5 MA and Volcano plots displaying differential gene expression between *Chromera*- infected and control samples at 4 h. MA plots for differential expression analysis for each gene, the log_2_ (fold change) between the two samples is plotted against the gene’s log_2_ (average expression) in the two samples. Volcano plots show the false discovery rate (-log_10_FDR) as a function of log_2_ (fold change). The red dots represent the significantly differentially expressed transcripts at adjusted *P* ≤ 0.05.

**
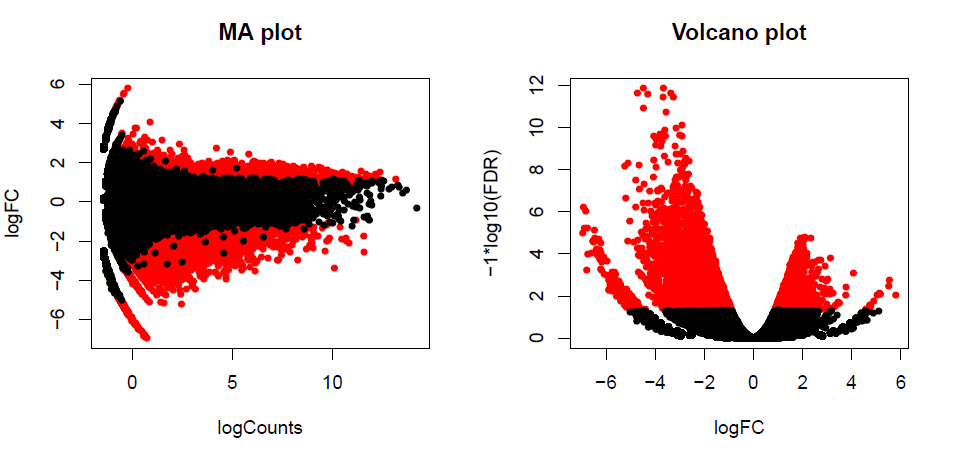
**

Figure S6 MA and Volcano plots displaying differential gene expression between *Chromera*-infected and control samples at 48 h. MA plots for differential expression analysis for each gene, the log_2_ (fold change) between the two samples is plotted against the gene’s log_2_ (average expression) in the two samples. Volcano plots show the false discovery rate (-log_10_FDR) as a function of log_2_ (fold change). The red dots represent the significantly differentially expressed transcripts adjusted to *P* < 0.05.

Figure S7 Differential gene expression profiles at 4 h post *Chromera* infection. The heat map shows the expression profiles of 48 DEGs in *Chromera*- infected versus control samples. The hierarchical clustering obtained by comparing the expression values (Fragments Per Kilobase of transcript per Million; FPKM) for *Chromera* infected samples compared against the control at 4h post infection. Expression values are log2-transformed and then median-centered by transcript. Relative expression levels are shown in red (up) and green (down). Samples I1, I2, I3 are the biological replicates of *Chromera* infection whilst samples C1, C2, C3 are the biological replicates of the control condition.

Figure S8 Differential gene expression profiles at 48 h post *Chromera* infection. The heat map shows the expression profiles of the 1086 highly differentially expressed genes (FDR<0.001, 4-fold) in *Chromera*- infected versus control samples. The hierarchical clustering obtained by comparing the expression values (Fragments Per Kilobase of transcript per Million; FPKM) for *Chromera* infected samples compared against the control at 48h post infection. Expression values are log2-transformed and then median-centered by transcript. Relative expression levels are shown in red (up) and green (down). Samples I1, I2, I3 are the biological replicates of *Chromera* infection whilst samples C1, C2, C3 are the biological replicates of the control condition.


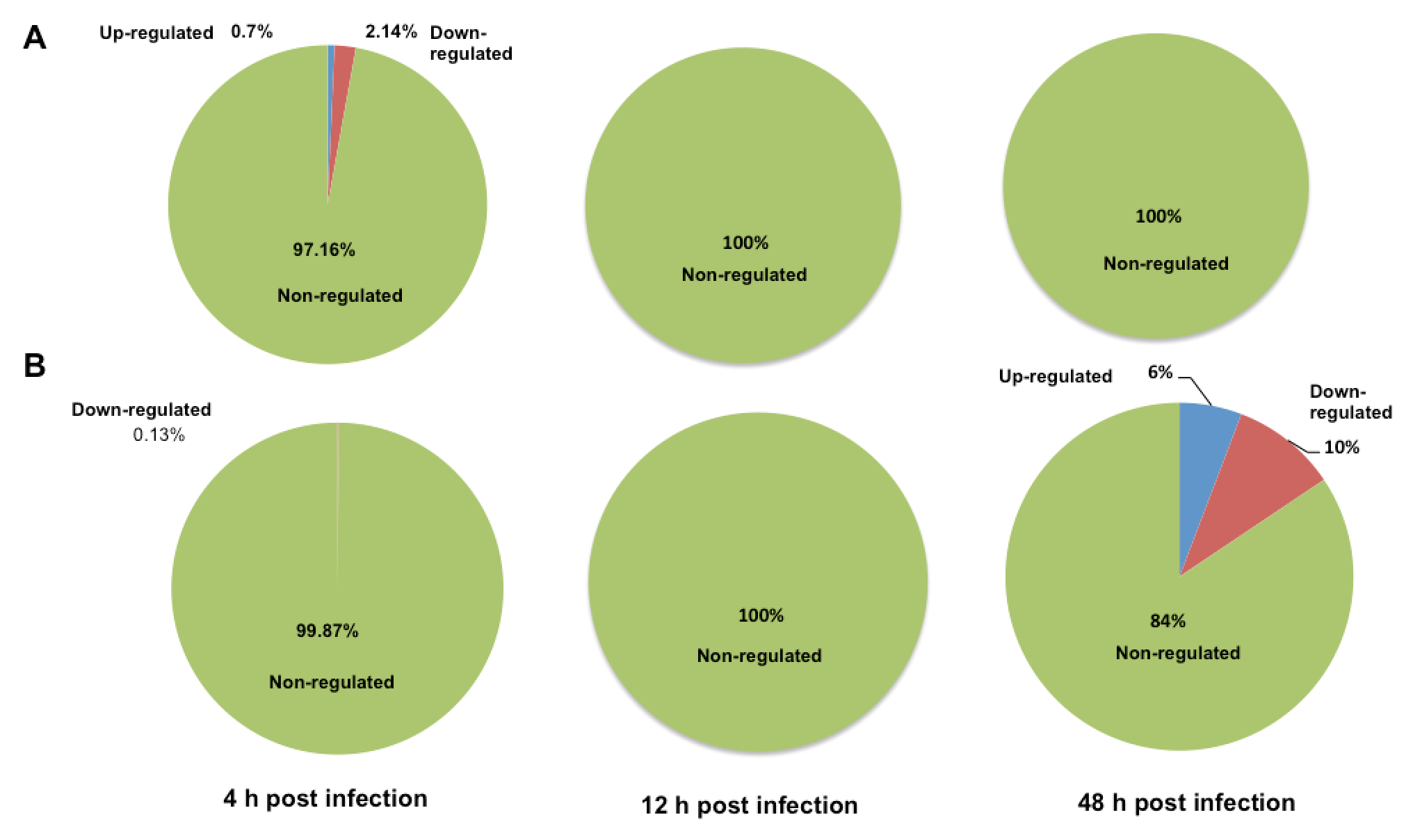


Figure S9 Comparison of *A. digitifera* transcriptome changes during competent *Symbiodinium* and *Chromera* infections. Panel A shows the transcriptome changes in *Symbiodinium*-infected larvae compared to control at 4, 12 and 48 h time points. Panel B shows the transcriptome changes in *Chromera*-infected larvae compared to control at 4, 12 and 48 h time points.

**
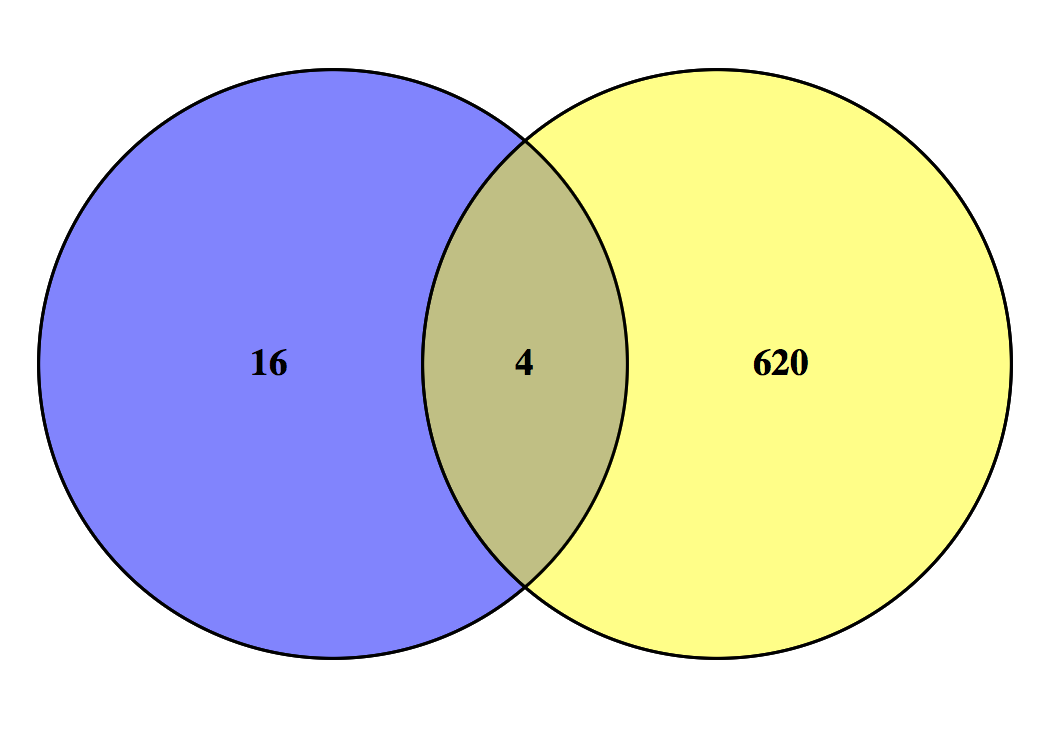
**

Figure S10 Venn diagram showing the overlap amongst significant *Acropora* DEGs aligned to the Swiss-Prot database common in *Chromera* (left; purple shading) and *Symbiodinium* (right; yellow shading; Mohamed *et al.,* 2016) infection compared to control at the 4 h time point.

**
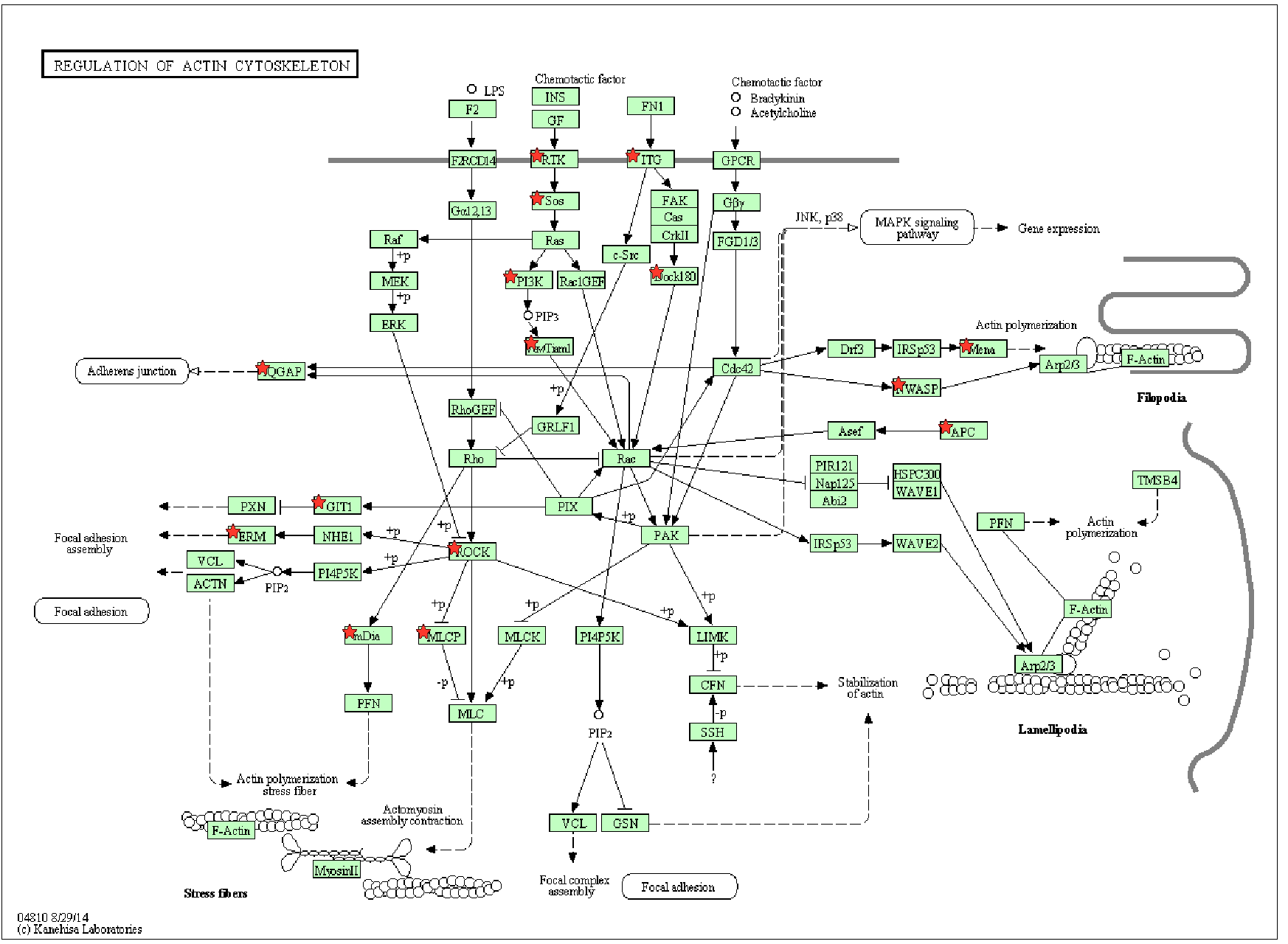
**

Figure S11 *Regulation of actin cytoskeleton­­* (KEGG pathway, hsa04810) genes significantly enriched in the set of down-regulated genes in *Chromera*-infected larvae at 48 h post infection. Red stars highlight proteins present in our dataset.

**
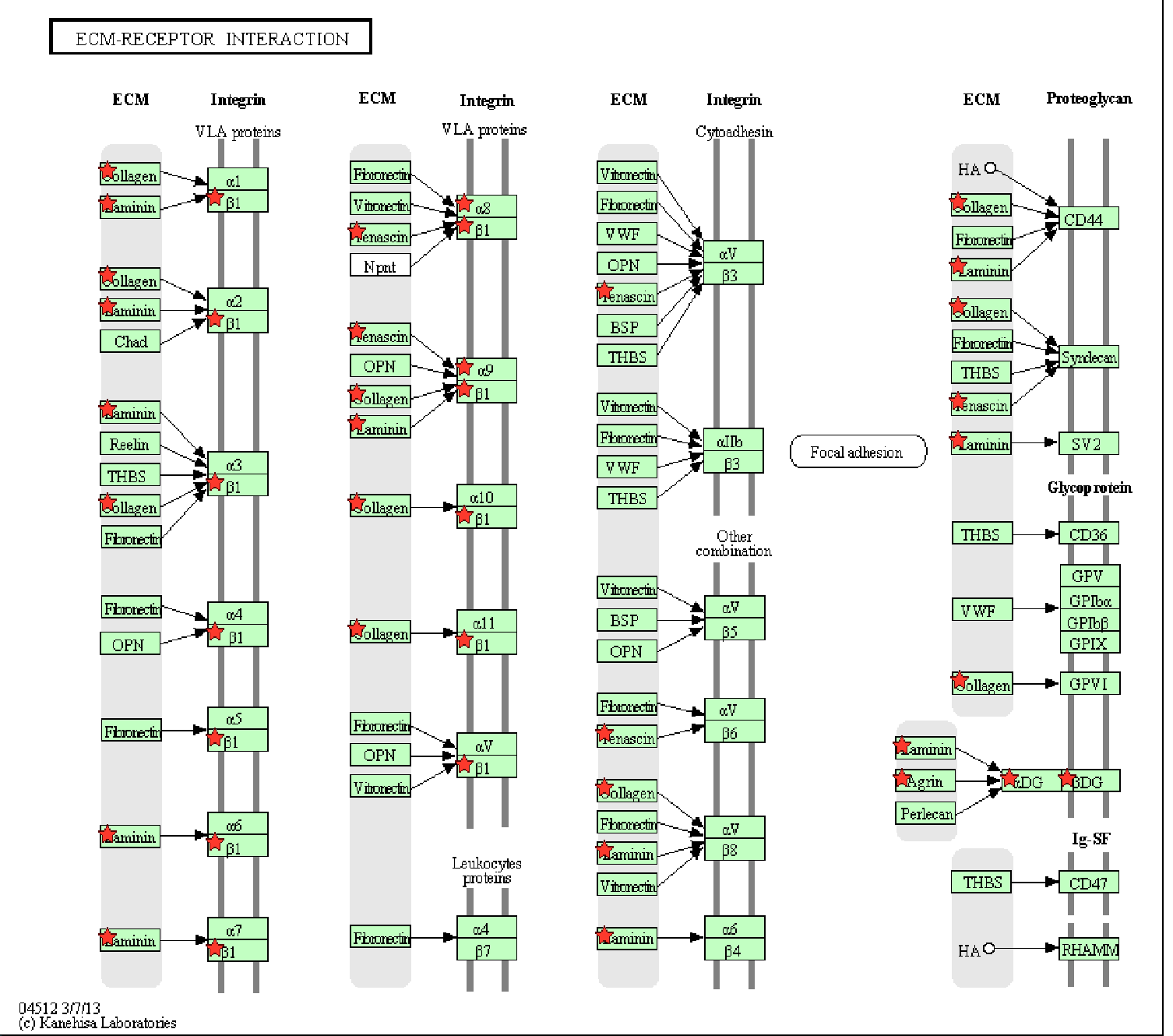
**

Figure S12 *ECM-receptor interaction* (KEGG pathway, hsa04512) genes significantly enriched in the set down-regulated in *Chromera*-infected larvae at 48 h post infection. Red stars highlight proteins present in our dataset.

**
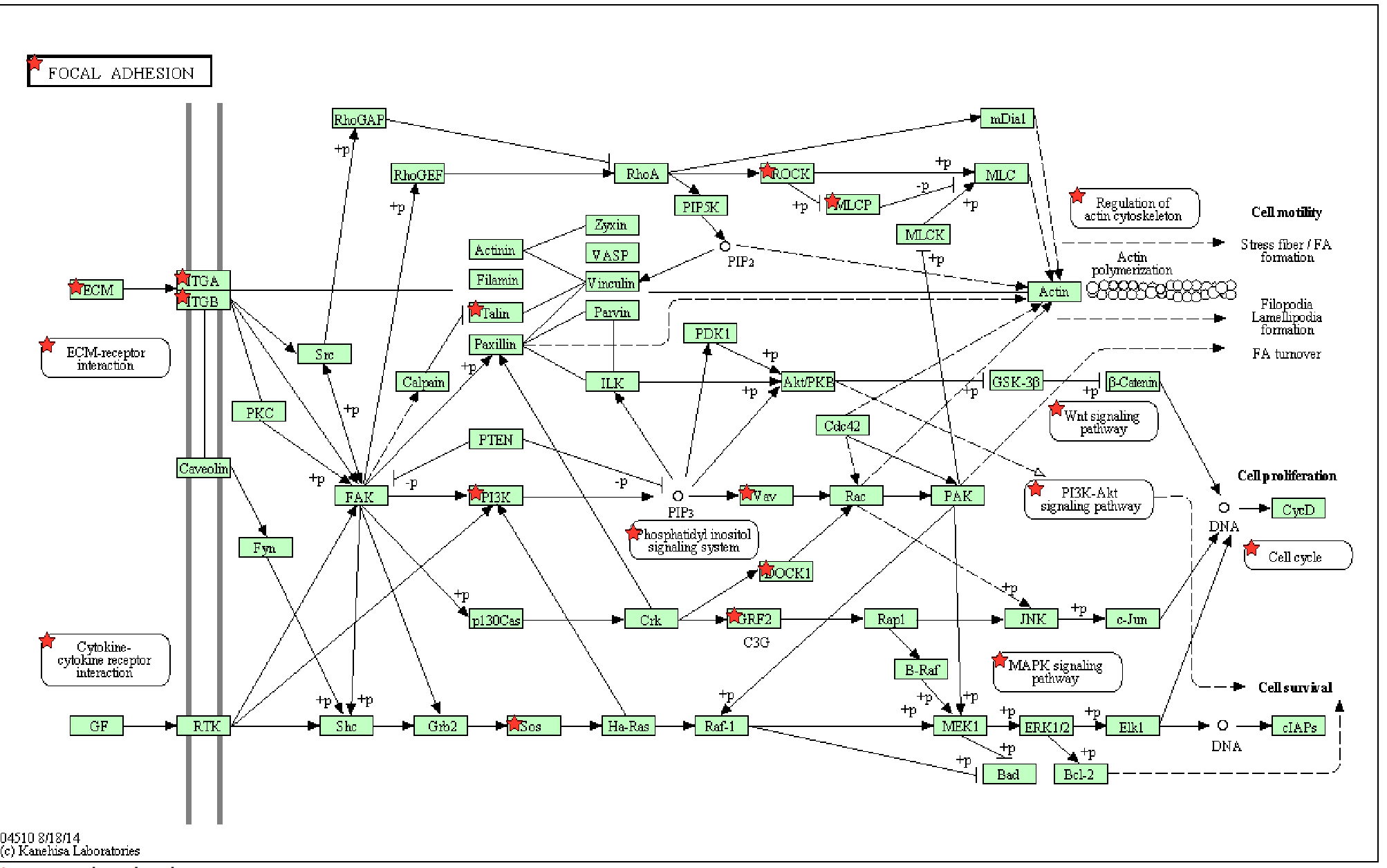
**

Figure S13 *Focal adhesion* (KEGG pathway, hsa04510) genes significantly enriched in the set of down-regulated in *Chromera*-infected larvae at 48 h post infection. Red stars highlight proteins present in our dataset.

**References**

Anders S, Huber W (2010). Differential expression analysis for sequence count data. *Genome biology* **11:** R106.

Huang da W, Sherman BT, Lempicki RA (2009). Systematic and integrative analysis of large gene lists using DAVID bioinformatics resources. *Nature protocols* **4:** 44-57.

Langmead B, Trapnell C, Pop M, Salzberg SL (2009). Ultrafast and memory-efficient alignment of short DNA sequences to the human genome. *Genome biology* **10:** R25.

Li B, Dewey CN (2011). RSEM: accurate transcript quantification from RNA-Seq data with or without a reference genome. *BMC bioinformatics* **12:** 323.

Robinson MD, McCarthy DJ, Smyth GK (2010). edgeR: a Bioconductor package for differential expression analysis of digital gene expression data. *Bioinformatics* **26:** 139-140.

Shinzato C, Shoguchi E, Kawashima T, Hamada M, Hisata K, Tanaka M *et al* (2011). Using the Acropora digitifera genome to understand coral responses to environmental change. *Nature* **476:** 320-323.

Thorvaldsdottir H, Robinson JT, Mesirov JP (2013). Integrative Genomics Viewer (IGV): high-performance genomics data visualization and exploration. *Briefings in bioinformatics* **14:** 178-192.
